# Sphingosine 1-phosphate receptor 5 (S1PR5) regulates the peripheral retention of tissue-resident lymphocytes

**DOI:** 10.1084/jem.20210116

**Published:** 2021-10-22

**Authors:** Maximilien Evrard, Erica Wynne-Jones, Changwei Peng, Yu Kato, Susan N. Christo, Raissa Fonseca, Simone L. Park, Thomas N. Burn, Maleika Osman, Sapna Devi, Jerold Chun, Scott N. Mueller, George Kannourakis, Stuart P. Berzins, Daniel G. Pellicci, William R. Heath, Stephen C. Jameson, Laura K. Mackay

**Affiliations:** 1 Department of Microbiology and Immunology, The University of Melbourne at The Peter Doherty Institute for Infection and Immunity, Melbourne, Victoria, Australia; 2 Center for Immunology, University of Minnesota Medical School, Minneapolis, MN; 3 Department of Laboratory Medicine and Pathology, University of Minnesota Medical School, Minneapolis, MN; 4 The ARC Centre of Excellence in Advanced Molecular Imaging, University of Melbourne, Parkville, Victoria, Australia; 5 Sanford Burnham Prebys Medical Discovery Institute, La Jolla, CA; 6 Federation University Australia and Fiona Elsey Cancer Research Institute, Ballarat, Victoria, Australia; 7 Cellular Immunology Group, Murdoch Children’s Research Institute, Melbourne, Victoria, Australia; 8 Department of Paediatrics, The University of Melbourne, Melbourne, Victoria, Australia

## Abstract

Tissue-resident memory T (T_RM_) cells provide long-lasting immune protection. One of the key events controlling T_RM_ cell development is the local retention of T_RM_ cell precursors coupled to downregulation of molecules necessary for tissue exit. Sphingosine-1-phosphate receptor 5 (S1PR5) is a migratory receptor with an uncharted function in T cells. Here, we show that S1PR5 plays a critical role in T cell infiltration and emigration from peripheral organs, as well as being specifically downregulated in T_RM_ cells. Consequentially, T_RM_ cell development was selectively impaired upon ectopic expression of *S1pr5*, whereas loss of *S1pr5* enhanced skin T_RM_ cell formation by promoting peripheral T cell sequestration. Importantly, we found that T-bet and ZEB2 were required for *S1pr5* induction and that local TGF-β signaling was necessary to promote coordinated *Tbx21*,* Zeb2*, and *S1pr5* downregulation. Moreover, S1PR5-mediated control of tissue residency was conserved across innate and adaptive immune compartments. Together, these results identify the T-bet–ZEB2–S1PR5 axis as a previously unappreciated mechanism modulating the generation of tissue-resident lymphocytes.

## Teaser

S1PR5 impairs T_RM_ cell differentiation by limiting T cell entry and promoting T cell egress from peripheral tissues. Local TGF-β signaling coordinates suppression of the T-bet–ZEB2–S1PR5 emigration axis, thus enforcing tissue residency.

## Graphical Abstract

**Figure:**
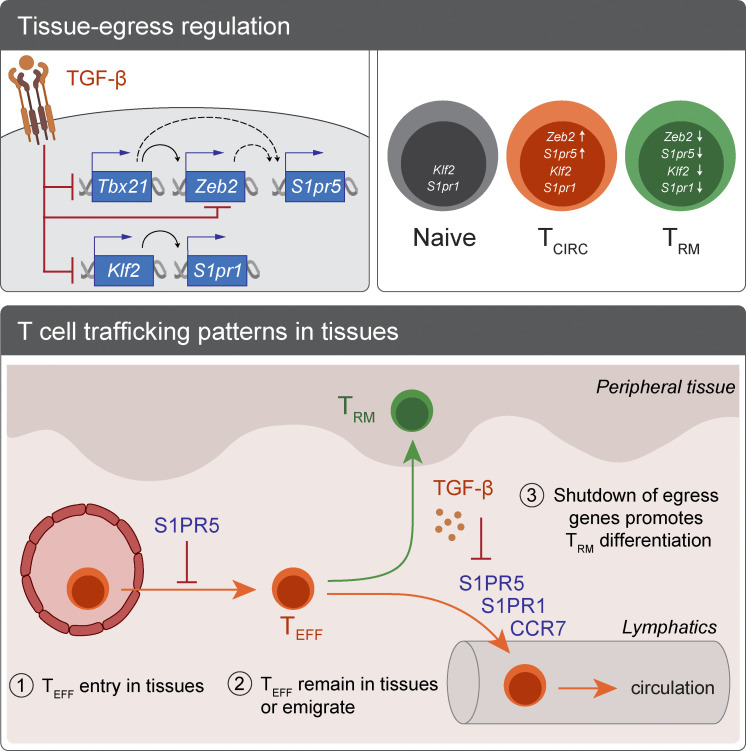

## Introduction

Effective immune protection relies on the generation of long-lived memory T cells. During a typical immune response, naive T cells surveying lymphoid organs are activated by cognate antigen and differentiate into short-lived effector cells (SLECs) or long-lived memory T cells (Kaech and Cui, 2012; Omilusik and Goldrath, 2019). Following their activation, T cells exit lymphoid organs and infiltrate infected tissues where they can mediate pathogen clearance. Memory T cells persist after infection resolution to provide protection against subsequent encounters with the same pathogen. These memory cells can be partitioned into distinct subsets based on their migratory and functional properties. Nonrecirculating tissue-resident memory T cells (T_RM_ cells) reside in a wide range of organs, such as the skin, gut, and lung. There, they form a defensive barrier, providing potent immune protection against a plethora of infections and cancer (Masopust and Soerens, 2019; Park et al., 2019; Szabo et al., 2019). After effector T cells enter inflamed tissues, multiple factors determine whether T_RM_ cell precursors “stay” to receive local signals that promote T_RM_ cell differentiation, or “go” and return to the circulation (Mackay et al., 2013). These decisions fundamentally shape the course of the immune response and the magnitude of protective immunity that ensues, yet the signals orchestrating these processes are not yet fully understood.

T_RM_ cell differentiation is not a default pathway for T cells entering peripheral tissues but is instead a multistep process that requires the infiltration, retention, and long-term survival of T cells in the tissue. At epithelial surfaces, local tissue recruitment is achieved via the guidance of effector T cells through chemokine receptors, such as CCR9, CXCR3, and CXCR6 (Mackay et al., 2013; Masopust et al., 2010; Takamura et al., 2019). In contrast, tissue egress of effector and memory T cells is facilitated via the lymphatic system that is rich in chemoattractants, including CCL19/CCL21 (recognized by CCR7; Bromley et al., 2005; Debes et al., 2005) and sphingosine 1-phosphate (S1P; Skon et al., 2013). The sphingolipid S1P is highly abundant in blood and lymph and can bind to five different receptors (S1PR1–5; Baeyens and Schwab, 2020; Ishii et al., 2004; Kihara et al., 2014). Among these receptors, S1PR1 controls key aspects of T cell trafficking from the migration of naive T cells out of LNs to the egress of effector T cells from peripheral organs (Matloubian et al., 2004; Skon et al., 2013). At the transcriptional level, S1PR1 expression is induced by Kruppel-like factor 2 (KLF2; Carlson et al., 2006), and downregulation of *Klf2* and *S1pr1* are essential for T_RM_ cell differentiation in a wide variety of organs (Skon et al., 2013). In addition, the activation-induced surface molecule CD69, which is found on most T_RM_ cells, can directly bind to S1PR1 and promote its degradation, thereby antagonizing tissue egress and bolstering T_RM_ cell differentiation in the skin (Mackay et al., 2015a; Shiow et al., 2006; Walsh et al., 2019).

While the role of S1PR1 in regulating T cell migration is well established, the contribution of the other S1PR family members is less clear (Baeyens and Schwab, 2020). Among S1PRs, we have previously observed that S1PR5 is expressed by memory CD8^+^ T cells and selectively downregulated in T_RM_ cells (Mackay et al., 2013, 2016). Although S1PR5 has no ascribed function in T cells, it has been reported that S1PR5 mediates natural killer (NK) cell migration, promoting NK cell egress from the bone marrow (BM) and LNs (Mayol et al., 2011; Walzer et al., 2007). Mice carrying a mutation in* Tbx21*, the gene encoding the T-box transcription factor expressed in T cells (T-bet), exhibit reduced S1PR5 expression in NK cells, suggesting that T-bet induces *S1pr5* (Jenne et al., 2009). In addition, unlike S1PR1, S1PR5 does not interact with CD69 (Jenne et al., 2009), together highlighting salient differences in the molecular regulation of these two receptors. Nonetheless, whether the control of S1PR5 expression is similar in CD8^+^ T cells and to what extent this receptor may regulate their trafficking remain unclear.

Here, we characterized the expression, regulation, and function of S1PR5 in effector and memory T cell subsets. We found that akin to S1PR1, downregulation of S1PR5 was required for efficient T_RM_ cell differentiation. However, in contrast to S1PR1, which is uniformly expressed in naive and circulating memory T cells under the control of KLF2, S1PR5 expression was only induced following antigen experience and was predominantly driven by the transcription regulator zinc finger E-box binding homeobox 2 (ZEB2), which acts downstream of T-bet. We found that tissue-derived TGF-β was necessary to promote the downregulation of *Tbx21* and *Zeb2*, and ultimately *S1pr5*, thereby hindering tissue traversion and promoting T_RM_ cell formation. Moreover, we identified a similar role for S1PR5 in potentiating the development of certain tissue-resident innate lymphoid cell (ILC) populations. Collectively, our study identifies S1PR5 as a novel regulator of T cell trafficking that governs formation of tissue-resident lymphocyte populations.

## Results

### Differential expression of S1PRs by circulating and resident CD8^+^ T cells

**Figure 1.**
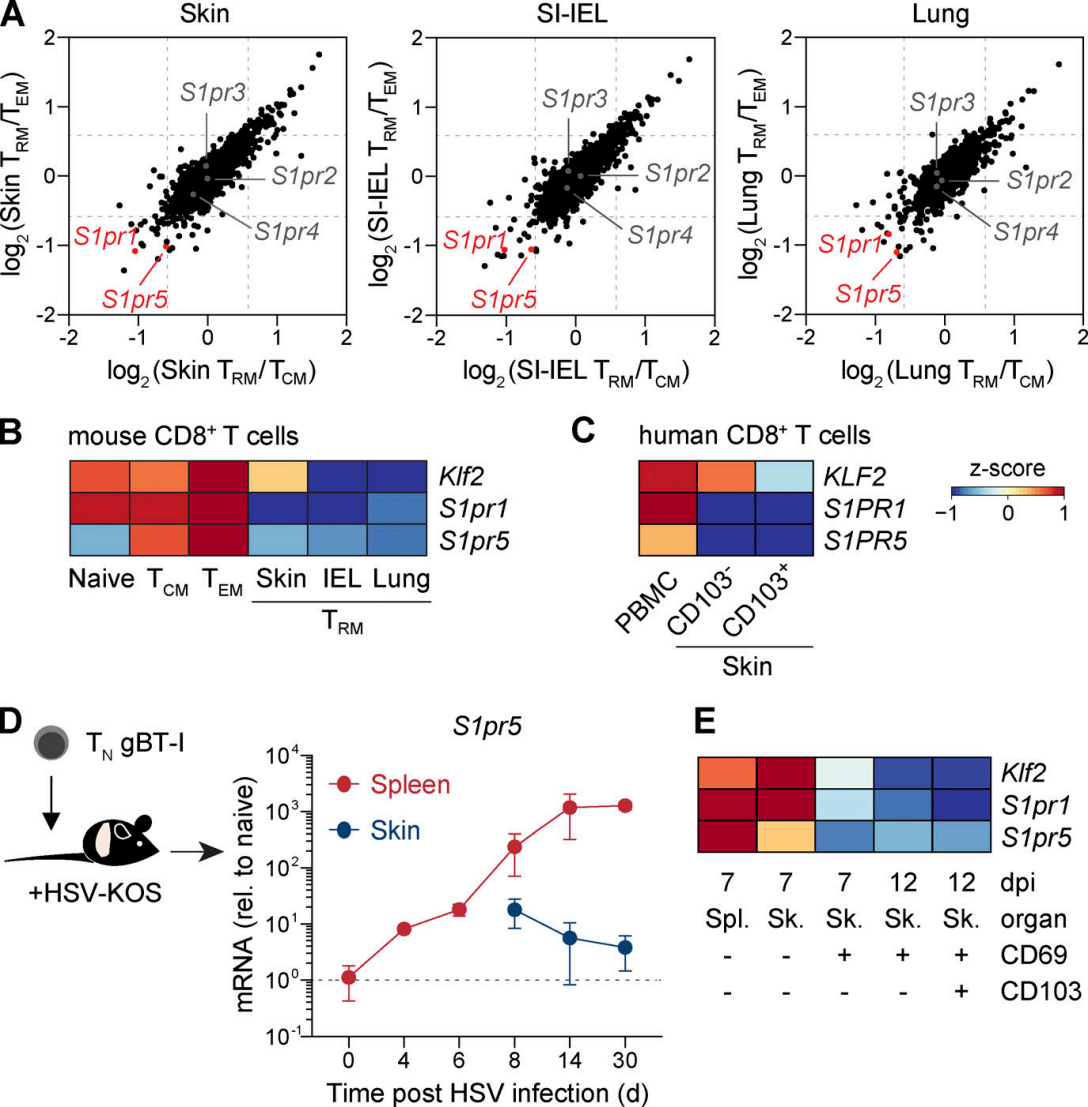
**S1PR5 expression during memory CD8^+^ T cell differentiation. (A)** Gene expression of skin, SI-IEL, or lung T_RM_ cells relative to splenic T_CM_ and T_EM_ cells >30 d after HSV (skin T_RM_), LCMV (intestine intraSI-IEL T_RM_), or WSN.gB (lung T_RM_) infection. Gene expression was extracted from the Mackay et al. (2013) dataset (GEO accession no. GSE47045). **(B and C)** Heatmap representations of gene expression (*z*-score normalized by row) of mouse and human CD8^+^ T cell subsets. **(B)** Gene expression of indicated mouse CD8^+^ T cell subsets was analyzed from the Mackay et al. (2013) dataset (GEO accession no. GSE47045) and plotted after log_2_ transformation. **(C)** Gene expression of human CD8^+^ T cell subsets analyzed by qPCR and plotted as a heatmap (*z*-score normalized by row). Memory T cells were identified as CD8^+^CD45RO^+^ or CD8^+^CD69^+^CD103^+/−^ in PBMCs or skin, respectively. **(D and E)** qPCR analysis of gBT-I T cells isolated from the spleen or skin at indicated time points following HSV infection. **(D)** Gene expression was normalized to naive gBT-I T cells. Graph shows mean ± SD. **(E)** Gene expression of mouse CD8^+^ T cell subsets analyzed by qPCR and plotted as a heatmap (*z*-score normalized by row). In D and E, data are from two independent experiments, with *n* = 5–10 mice per time point. rel., relative; Sk., skin; Spl., spleen; T_N_, naive T cell.

To dissect the roles of the different S1PRs in memory T cell generation, we first examined the pattern of S1PR expression in T_RM_ cells and circulating memory T cell subsets. We began by comparing established transcriptomic profiles (Mackay et al., 2013) of antigen-specific CD69^+^CD103^+^CD8^+^ T_RM_ cells isolated from the skin, lung, and small intestine after HSV-KOS, influenza virus (WSN.gB), or acute lymphocytic choriomeningitis virus (LCMV-Armstrong) infection, respectively, with their circulating effector memory T cell (T_EM_ cell) and central memory T cell (T_CM_ cell) counterparts. Whereas *S1pr2*, *S1pr3*, and *S1pr4* were similarly expressed by resident and circulating T cell populations, *S1pr1* and *S1pr5* were selectively downregulated in T_RM_ cells across all three tissues (Fig. 1 A). Consistent with the known role for KLF2 in driving *S1pr1* expression (Carlson et al., 2006), *Klf2* and *S1pr1* transcripts were concordantly elevated in splenic naive T cells and T_EM_ and T_CM_ cells compared with T_RM_ cells (Fig. 1 B). In contrast, *S1pr5* was not expressed by naive CD8^+^ T cells (Fig. 1 B), suggesting that S1PR5 was likely regulated by factors other than KLF2. Analysis of analogous human CD8^+^ T cell populations revealed that *S1PR5*,* KLF2*, and *S1PR1* were likewise extinguished in CD103^+^ skin T_RM_ cells compared with circulating memory T cells in peripheral blood (Fig. 1 C), implying similar regulation of S1PRs in mouse and human CD8^+^ T cells.

To determine the kinetics of *S1pr5* expression during memory T cell differentiation, we transferred congenically marked CD45.1^+^ gBT-I transgenic CD8^+^ T cells specific for the immunodominant HSV epitope (gB_498–505_) into C57BL/6 mice before HSV skin infection. While *S1pr5* was not detected in naive gBT-I T cells, its expression was induced in splenic effector T cells as early as 4 d postinfection (dpi), increased over time, and was then maintained in circulating memory T cells for at least 30 d thereafter. Conversely, *S1pr5* expression diminished upon T cell entry into the skin, with expression continuing to decline between 14 and 30 dpi (Fig. 1 D). We found that *S1pr5* expression was extinguished before the upregulation of the integrin CD103 (Fig. 1 E), which is expressed following epidermal entry and indicative of full acquisition of the T_RM_ cell program (Mackay et al., 2015b). Taken together, our data indicate that akin to S1PR1, S1PR5 is an S1PR expressed by circulating effector and memory T cells that is downregulated during T_RM_ cell differentiation.

### T-bet cooperates with ZEB2 to regulate S1PR5 expression in CD8^+^ T cells

**Figure S1.**
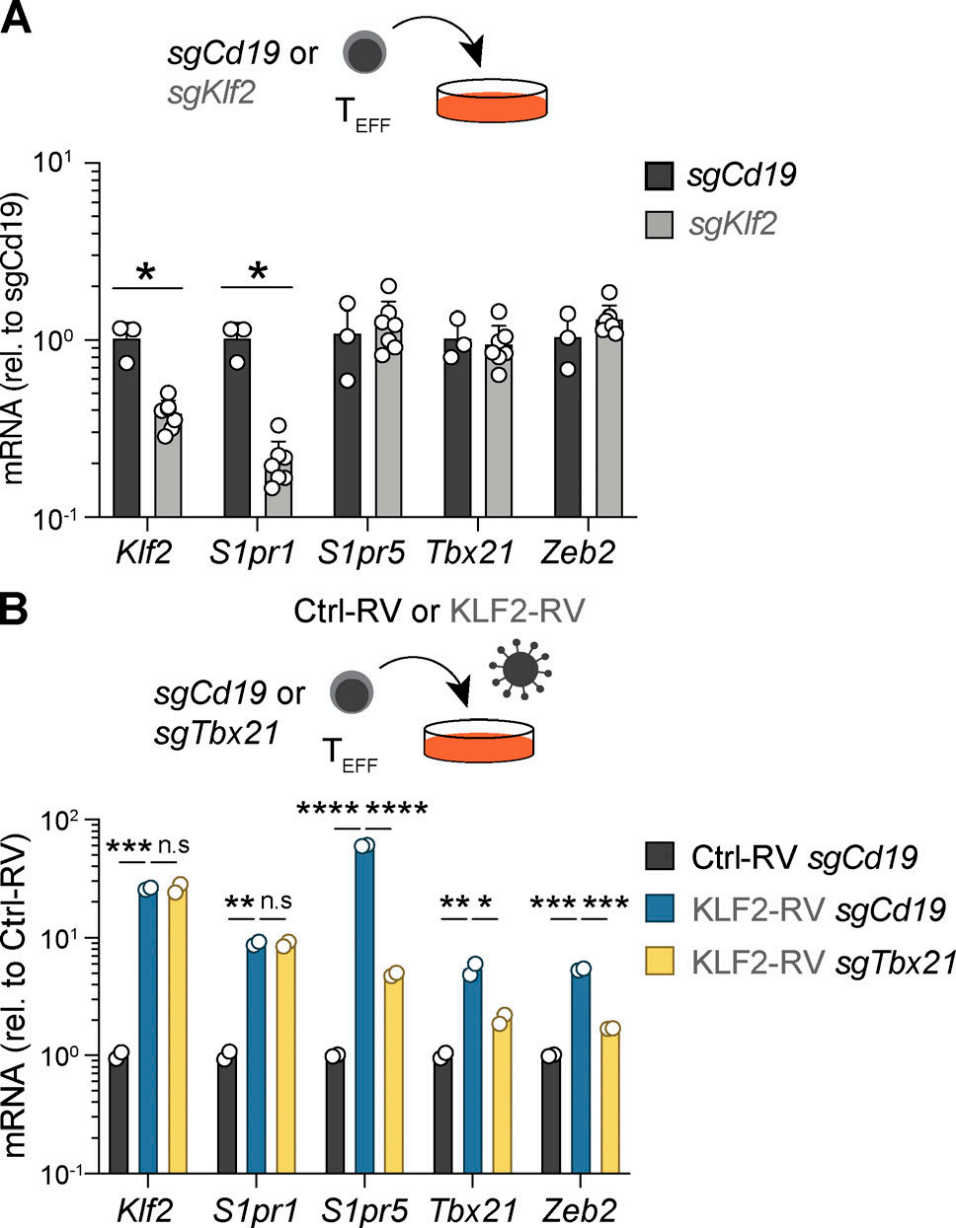
**KLF2 does not directly regulate *S1pr5* expression. (A and B)** Effector P14 T cells were nucleofected with control *Cd19*-targeting (*sgCd19*), *Klf2*-targeting (*sgKlf2*), or *Tbx21*-targeting (*sgTbx21*) sgRNA/Cas9 RNPs and were maintained in culture for 3 d. **(B)** Effector P14 T cells were transduced with a control (Ctrl-RV) or a KLF2 (KLF2-RV) R (RVs) before nucleofection with sgRNA/Cas9 RNPs. Expression of the indicated genes was quantified by qPCR. In A and B, data are pooled from two independent experiments, with *n* = 2–6 samples per condition. Multiple Mann–Whitney test was used in A and multiple *t* test in B. n.s., nonsignificant; *, P < 0.05; **, P < 0.01; ***, P < 0.001; ****, P < 0.0001. rel., relative; T_EFF_, effector T cell.

Discordant patterns of S1PR5 and S1PR1 expression in naive T cells suggested a differential role of KLF2 in regulating S1PR1 and S1PR5. To examine this, we first employed ribonucleoprotein (RNP)-based CRISPR/Cas9 technology (Nüssing et al., 2020; Seki and Rutz, 2018) to disrupt *Klf2* in in vitro–activated CD8^+^ T cells. While KLF2-ablated cells showed reduced *S1pr1* expression in agreement with previous studies (Carlson et al., 2006; Takada et al., 2011), *S1pr5* expression remained unchanged (Fig. S1 A). Interestingly, although KLF2 ablation did not affect *S1pr5* transcript levels, overexpression of KLF2 in CD8^+^ T cells via retroviral transduction increased both *S1pr1* and *S1pr5* in vitro (Fig. S1 B). Previous studies have shown that forced KLF2 expression is sufficient to induce T-bet in CD4^+^ T cells (Lee et al., 2015) and that S1PR5 expression in NK cells is controlled by T-bet (Jenne et al., 2009), implying an indirect link between KLF2 and S1PR5. Indeed, ablation of T-bet in KLF2-overexpressing cells markedly reduced *S1pr5* mRNA levels without changing *S1pr1* expression, demonstrating that S1PR5 is upregulated via T-bet and that KLF2 does not directly drive S1PR5 (Fig. S1 B).

**Figure 2.**
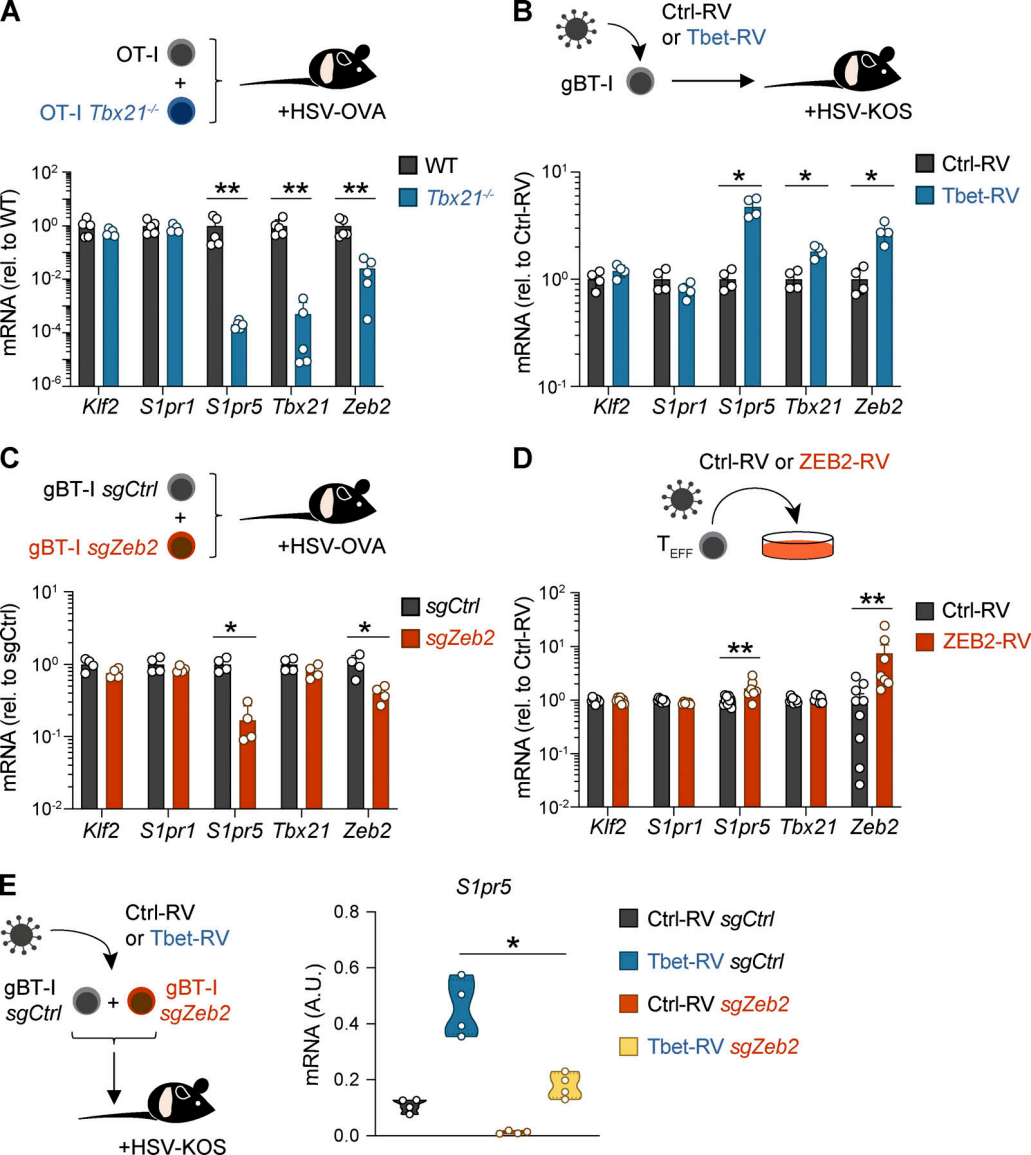
**T-bet and ZEB2-mediated control of S1PR5 expression in CD8^+^ T cells. (A)** Mice were adoptively transferred with naive GFP^+^ OT-I (OT-I WT) and CD45.1^+^ OT-I *Tbx21^−/−^* (OT-I *Tbx21*^−/−^) and infected with HSV-OVA. Expression of indicated genes quantified by qPCR on OT-I T cells from the spleen 8 dpi. **(B)** Effector gBT-I T cells were transduced with Ctrl-RV or Tbet-RV GFP-expressing RVs and cotransferred into mice infected with HSV. Expression of indicated genes quantified by qPCR on sort-purified gBT-I T cells from the spleen 8 dpi. **(C)** Effector gBT-I T cells were nucleofected with control-nontargeting (CD45.1^+^CD45.2^+^ gBT-I *sgCtrl*) or *Zeb2*-targeting (CD45.1^+^ gBT-I *sgZeb2*) sgRNA/Cas9 RNPs and cotransferred into HSV-infected mice. Expression of indicated genes quantified by qPCR on sort-purified transgenic T cells from the spleen 8 dpi. **(D)** Effector gBT-I T cells were transduced with control or ZEB2 (ZEB2-RV) RVs and maintained in culture with IL-15 for 3 d. Expression of indicated genes was quantified by qPCR in GFP^+^ gBT-I T cells. **(E)** Effector gBT-I T cells were transduced with control or Tbet-RV and nucleofected with control-nontargeting (CD45.1^+^CD45.2^+^ gBT-I *sgCtrl*) or *Zeb2*-targeting (CD45.1^+^ gBT-I *sgZeb2*) sgRNA/Cas9 RNPs and cotransferred into HSV-infected mice. *S1pr5* expression was quantified by qPCR in sort-purified gBT-I T cells from the spleen 8 d after transfer. In A–C and E, data are representative of two independent experiments, with *n* = 4–5 mice per experiment. In D, data are pooled from two to three independent experiments, with *n* = 2–3 samples per condition. *, P < 0.05, **, P < 0.01 by Mann–Whitney test. A.U., arbitrary units; rel., relative.

**Figure S2.**
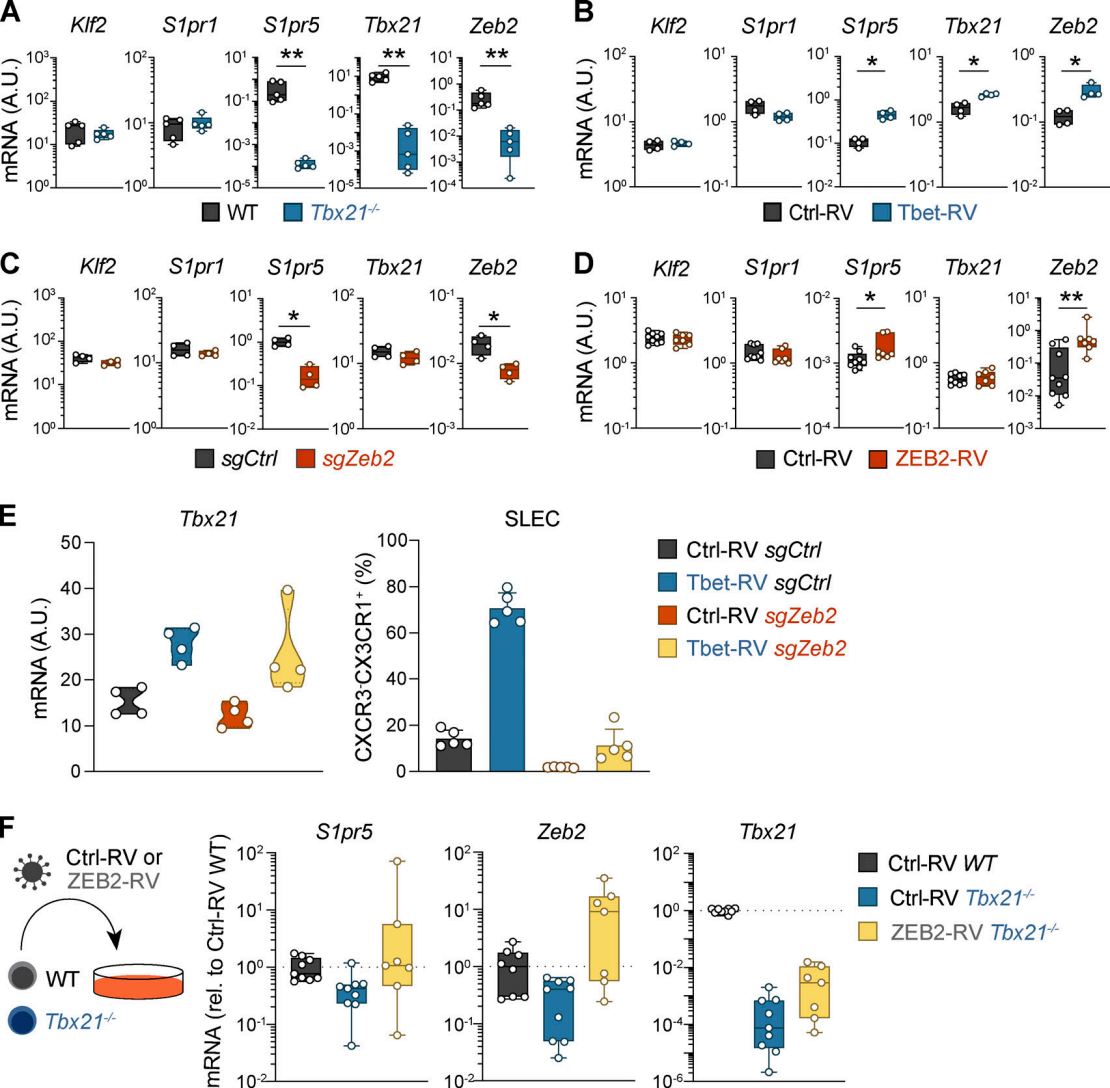
**Transcriptional control of SLEC CD8^+^ T cell differentiation and *S1pr5* expression via T-bet and Zeb2.** **(A)** Mice were adoptively transferred with naive GFP^+^ OT-I (OT-I WT) and CD45.1^+^ OT-I *Tbx21^−/−^* (OT-I *Tbx21*^−/−^) and infected with HSV-OVA. Expression of indicated genes quantified by qPCR on OT-I T cells from the spleen 8 dpi and shown normalized to a housekeeping gene. **(B)** Effector gBT-I T cells were transduced with control (Ctrl-RV) or T-bet (Tbet-RV) GFP-expressing RVs and cotransferred into mice infected with HSV. Expression of indicated genes quantified by qPCR on sort-purified gBT-I T cells from the spleen 8 dpi and shown normalized to a housekeeping gene. **(C)** Effector gBT-I T cells were nucleofected with control-nontargeting (CD45.1^+^CD45.2^+^ gBT-I *sgCtrl*) or *Zeb2*-targeting (CD45.1^+^ gBT-I *sgZeb2*) sgRNA/Cas9 RNPs and cotransferred into HSV-infected mice. Expression of indicated genes quantified by qPCR on sort-purified transgenic T cells from the spleen 8 dpi and shown normalized to a housekeeping gene. **(D)** Effector gBT-I T cells were transduced with control or ZEB2 (ZEB2-RV) RVs and maintained in culture with IL-15 for 3 d. Expression of indicated genes was quantified by qPCR in GFP^+^ gBT-I T cells and shown normalized to a housekeeping gene. **(E)** Ctrl-RV– or Tbet-RV–transduced gBT-I T cells were nucleofected with control nontargeting (*sgCtrl*) or *Zeb2* targeting (*sgZeb2*) sgRNA/Cas9 RNPs and cotransferred into HSV-infected mice. gBT-I T cells were sort-purified from the spleen 8 d after transfer. *Tbx21* expression was quantified by qPCR and shown normalized to a housekeeping gene (left), and frequencies of SLECs were analyzed by flow cytometry (right). **(F)** Effector OT-I WT of *Tbx21^−/−^* were transduced with Ctrl-RV or ZEB2-RV and cultured with IL-15 for 3 d. Expression of the indicated genes in transduced cells was quantified by qPCR. In A–E, data are representative of two independent experiments, with *n* = 4–5 mice per group per experiment. In F, data are pooled from three independent experiments. *, P < 0.05; **, P < 0.01 by Mann–Whitney test. Graph shows mean ± SD. A.U., arbitrary units; rel., relative.

To investigate the role of T-bet in governing S1PR5 expression in vivo, we cotransferred congenically distinct naive OT-I transgenic CD8^+^ T cells specific for OVA (OVA_257–264_) and T-bet–deficient OT-I T cells (OT-I *Tbx21^−/−^*) into naive C57BL/6 mice before infection with a recombinant strain of HSV-expressing OVA (HSV-OVA). *S1pr5* expression was abolished in T-bet-deficient OT-I T cells in the spleen 8 dpi, while *S1pr1* and *Klf2* expression remained unchanged (Figs. 2 A and S2 A). Furthermore, forced expression of T-bet by retroviral transduction in HSV-primed gBT-I T cells led to *S1pr5* upregulation with no impact on *Klf2* and *S1pr1* expression (Figs. 2 B and S2 B). T-bet can promote the expression of transcription factors, including ZEB2, which can in turn regulate multiple gene targets to coordinate lymphocyte differentiation (Dominguez et al., 2015; Omilusik et al., 2015; van Helden et al., 2015). Consistent with this, we observed *Zeb2* upregulation following retrovirus (RV)-induced T-bet expression (Figs. 2 B and S2 B) and reduced expression of *Zeb2* in T-bet–deficient T cells responding to HSV infection, which correlated with a marked decrease in *S1pr5* expression (Figs. 2 A and S2 A). We therefore asked whether S1PR5 was directly regulated by T-bet or induced indirectly via ZEB2. To this end, we used CRISPR/Cas9 to ablate *Zeb2* in effector gBT-I T cells before transfer into HSV-infected mice. Critically, *S1pr5* transcripts were substantially reduced in ZEB2-ablated cells, despite similar expression of *Tbx21*,* Klf2*, and *S1pr1* compared with control T cells (Figs. 2 C and S2 C). This was in agreement with data indicating reduced T-bet binding to *S1pr5* promoter in the absence of ZEB2 (Dominguez et al., 2015). Conversely, forced expression of ZEB2 in effector CD8^+^ T cells using retroviral transduction was sufficient to drive *S1pr5* upregulation in vitro (Figs. 2 D and S2 D). Hence, ZEB2 appeared to be the primary regulator of *S1pr5* in CD8^+^ T cells, with T-bet acting upstream of ZEB2. To corroborate this, we forced T-bet expression in ZEB2-ablated or control gBT-I T cells via retroviral transduction and transferred these cells into HSV-infected mice. While T-bet overexpression in gBT-I T cells potentiated SLEC formation, the differentiation of SLEC was reduced when T-bet expression was forced in ZEB2-ablated gBT-I T cells (Fig. S2 E), in agreement with the known role of ZEB2 in driving SLEC differentiation (Dominguez et al., 2015; Omilusik et al., 2015). Importantly, whereas forced T-bet expression increased *S1pr5* expression in control gBT-I T cells, this effect was greatly diminished in the absence of ZEB2, although T-bet could still promote low levels of *S1pr5* in ZEB2-ablated cells (Fig. 2 E). In contrast, ZEB2 overexpression in *Tbx21^−/−^* cells was sufficient to maintain *S1pr5* expression in vitro (Fig. S2 F), reminiscent of observations in NK cells (van Helden et al., 2015). Overall, these findings support a model whereby T-bet indirectly regulates *S1pr5* by activating expression of ZEB2, which then acts as the major proponent of *S1pr5* induction.

### Forced expression of S1PR5 perturbs T cell localization in secondary lymphoid organs

**Figure S3.**
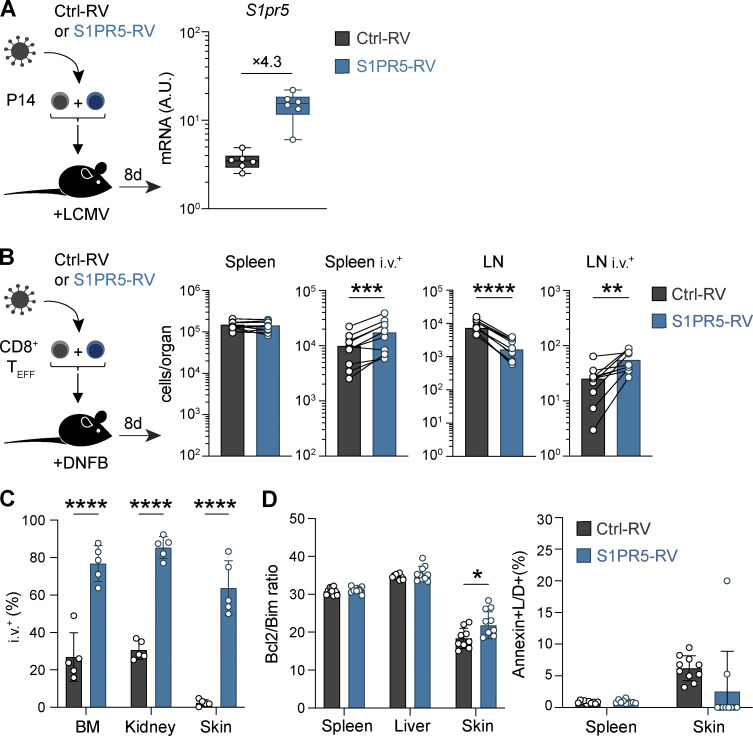
**S1PR5 promotes T cell relocation to vascular beds. (A)** P14 T cells were transduced with control (Ctrl)-RV or S1PR5-RV and transferred into LCMV-infected mice. Transduced cells were sorted from the spleen 8 dpi. Expression of *S1pr5* was quantified by qPCR and normalized to a housekeeping gene. **(B–D)** OT-I T cells were transduced with Ctrl-RV or S1PR5-RV and cotransferred i.v. into mice treated with DNFB. Mice received anti-CD45 antibody i.v. to label vasculature-associated cells before harvest. Shown are numbers (B) and frequencies (C) of Ctrl-RV and S1PR5-RV cells isolated from the indicated organs 8 d after transfer and the Bcl2/Bim ratio (left) and percentage annexin^+^ live/dead^+^ (L/D) apoptotic cells (right) of Ctrl-RV and S1PR5-RV cells isolated from the indicated organs 8 d after transfer (D). In A, data are pooled from two independent experiments, with *n* = 3 mice per group per experiment. In B–D, data are representative of two independent experiments, with *n* = 5–10 mice per experiment. Multiple paired *t* test was used in B and multiple unpaired *t* test in C and D. *, P < 0.05; **, P < 0.01; ***, P < 0.001; ****, P < 0.0001. Graph shows mean ± SD. A.U., arbitrary units; T_EFF_, effector T cell.

**Figure 3.**
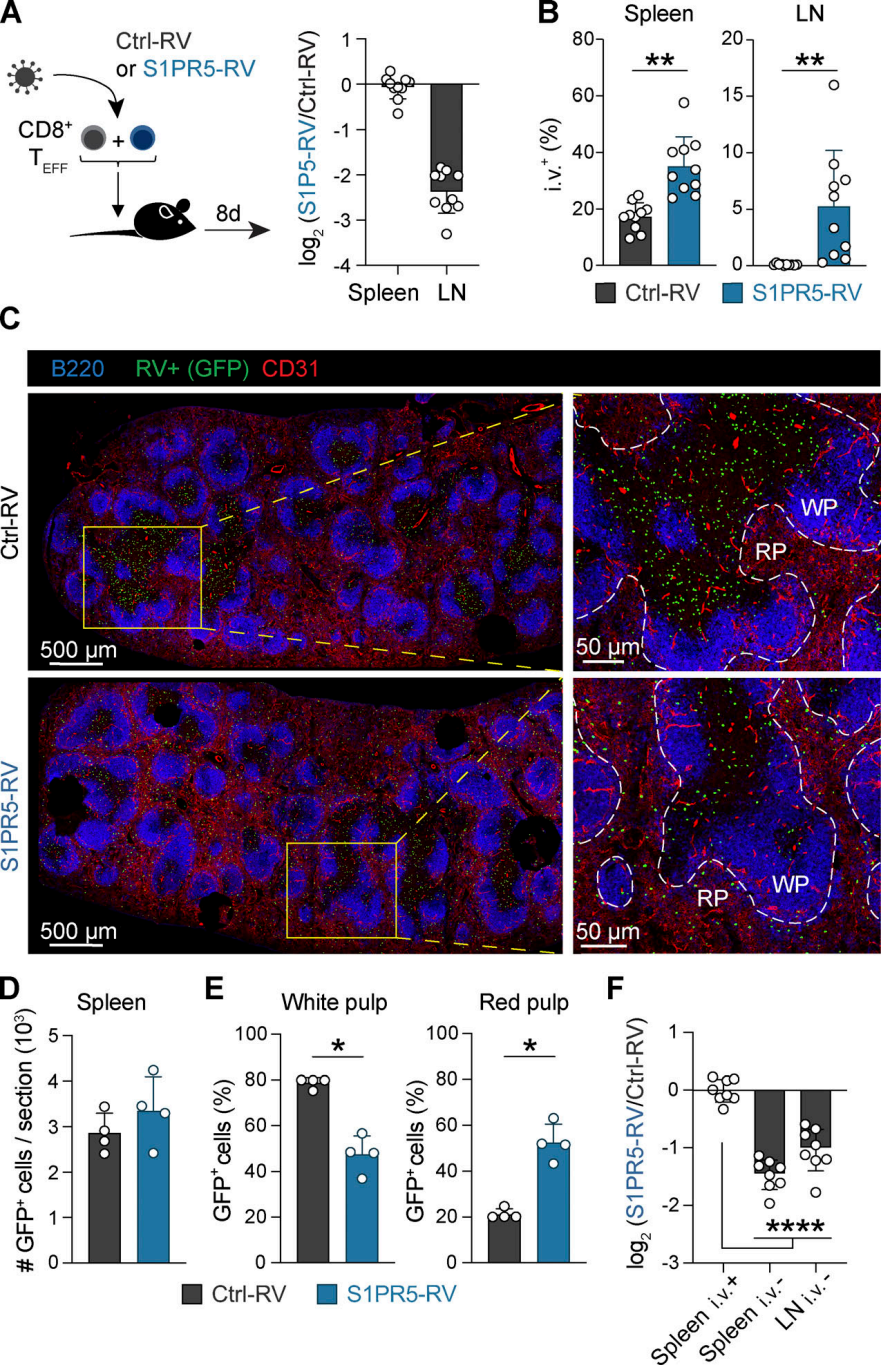
**S1PR5 expression alters CD8^+^ T cell trafficking in lymphoid organs. (A and B)** Effector OT-I T cells carrying distinct congenic markers were transduced with control (Ctrl-RV) or S1PR5 (S1PR5-RV) GFP-expressing RVs and cotransferred into recipient mice. Transduced cells were isolated from the spleen and inguinal LNs 8 d after transfer. **(A)** Ratio of Ctrl-RV and S1PR5-RV cells isolated from indicated organs is shown. **(B)** Mice were administered i.v. with anti-CD45 antibody (i.v.^+^) to label vasculature-associated cells. Frequencies of i.v.^+^ Ctrl-RV and S1PR5-RV cells from indicated organs are shown. **(C–E)** Effector CD45.1^+^ gBT-I T cells were transduced with control or S1PR5 RVs and transferred into recipient mice. Spleens were harvested 7 d after transfer. **(C)** Confocal images of the spleen showing the localization of GFP^+^ Ctrl-RV or S1PR5-RV cells (green) in relation to B cells (B220^+^, blue) and endothelial cells (CD31^+^, red). Selected areas are magnified (right panels) to highlight the boundary between WP and RP. Lack of CD31 staining was used to delineate WP/RP separation (dotted lines). **(D)** Number of GFP^+^ cells on whole spleen sections were quantified. **(E)** Frequencies of Ctrl-RV and S1PR5-RV cells located in the WP and RP were calculated. *n* = 4 mice per group. **(F)** Effector OT-I T cells carrying distinct congenic markers were transduced with Ctrl-RV or S1PR5-RV, cotransferred into recipient mice, and isolated from the spleen and LNs 2 h later. Mice were i.v. labeled before harvest. Ratio of Ctrl-RV and S1PR5-RV cells isolated from the indicated organs were normalized to splenic i.v.^+^ cells. In A, B, and F, data are representative of two independent experiments, with *n* = 8–10 mice per experiment. Wilcoxon test was used in B, Mann–Whitney test in E, and one-way ANOVA in F. *, P < 0.05; **, P < 0.01; ****, P < 0.0001. Graph shows mean ± SD. T_EFF_, effector T cell.

S1PR5 upregulation is required for NK cell trafficking from the BM and LNs to the periphery (Jenne et al., 2009; Mayol et al., 2011; Walzer et al., 2007). However, a potential role for S1PR5 in regulating T cell migration has not been explored. To this end, we used RVs to drive S1PR5 expression in CD8^+^ T cells, which yielded a fourfold increase in *S1pr5* gene expression compared with effector CD8^+^ T cells primed by LCMV infection (Fig. S3 A). To test the impact of S1PR5 expression on CD8^+^ T cell migration, we transduced congenically marked effector CD8^+^ T cells with S1PR5 (S1PR5-RV) or control (Ctrl-RV) RVs and cotransferred these cells into naive mice. While forced expression of S1PR5 did not appear to influence T cell localization to the spleen, S1PR5-RV cells were severely underrepresented in LNs (Fig. 3 A). We hypothesized that the discrepancy between S1PR5 control of spleen and LN localization might be attributed to distinct tissue architecture in each lymphoid organ, with the spleen being highly vascularized compared with LNs. To address this, we performed intravascular labeling to distinguish cells located within blood vessels from those in the tissue parenchyma (Anderson et al., 2014). Relative to Ctrl-RV cells, S1PR5-RV cells exposed to the vasculature were increased in both number and frequency (Fig. 3 B and Fig. S3, B and C). Immunofluorescence staining confirmed that while similar numbers of Ctrl-RV and S1PR5-RV cells were observed in the spleen, the majority of Ctrl-RV cells localized to the T cell zone of the white pulp (WP), whereas S1PR5-expressing cells redistributed to the red pulp (RP) at higher frequencies (Fig. 3, C–E).

While S1PR5 has been described to drive cellular egress from lymphoid tissues (Jenne et al., 2009), it remained unclear whether S1PR5 expression could also limit T cell entry into tissues. To investigate this, we analyzed the ability of S1PR5-RV and Ctrl-RV cells to migrate to the spleen and LN 2 h after cell transfer and observed a reduction of S1PR5-RV in the splenic WP and LN parenchyma (Fig. 3 F). Overall, these findings indicate that S1PR5 expression can hinder the entry of CD8^+^ T cells in lymphoid tissues such as LNs and spleen WP and promote their relocalization into vascular beds.

### S1PR5 expression controls bidirectional tissue trafficking and impairs T_RM_ cell formation

**Figure 4.**
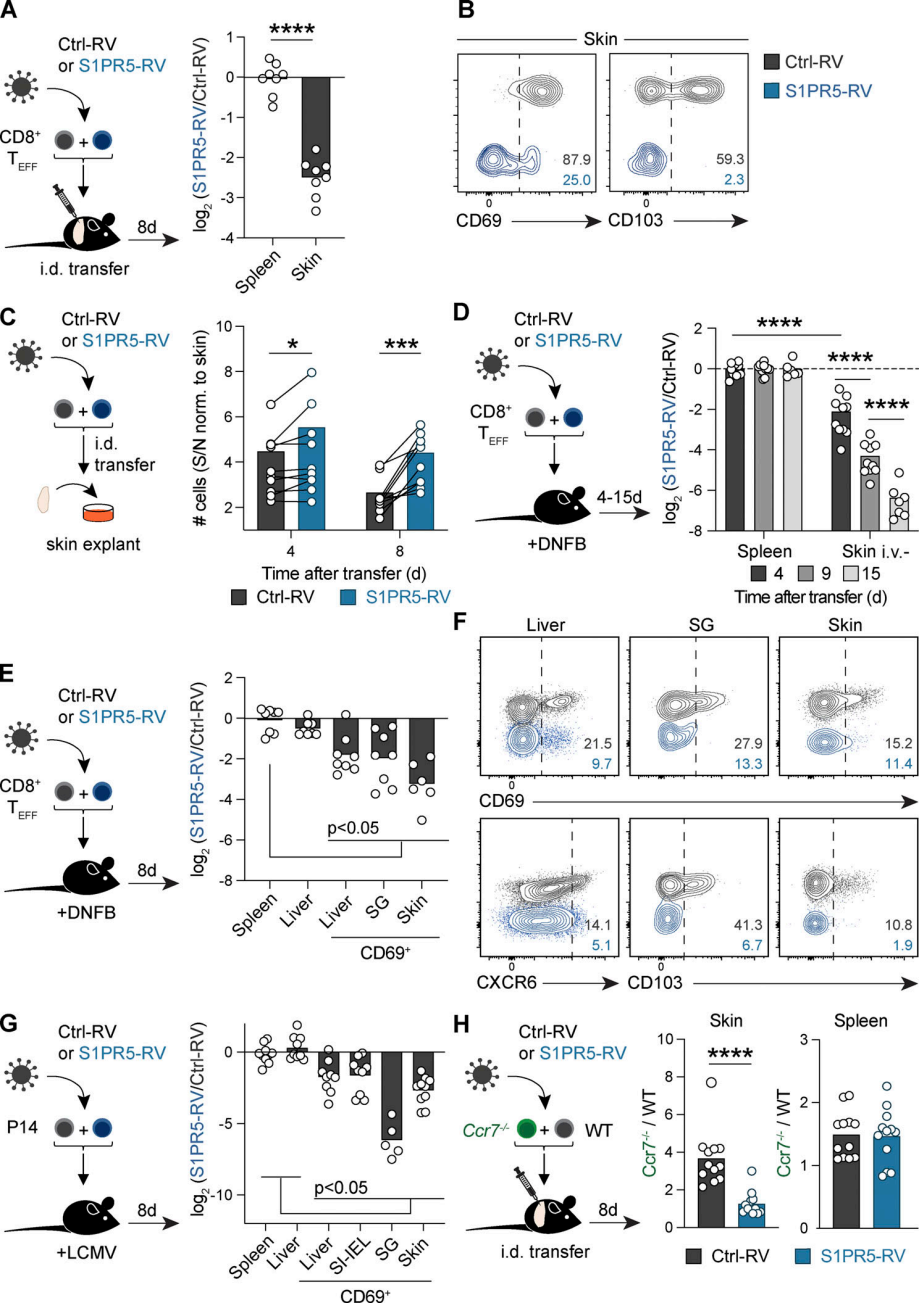
**S1PR5 induces CD8^+^ T cell egress from nonlymphoid tissues. (A–C)** Effector gBT-I T cells carrying distinct congenic markers were transduced with Ctrl-RV or S1PR5-RV and cotransferred intradermally (i.d.) into the flank of naive mice. **(A)** Ratios of Ctrl-RV and S1PR5-RV cells isolated from the indicated organs normalized to those in the spleen. **(B)** CD69 and CD103 expression on Ctrl-RV or S1PR5-RV cells isolated from the skin 8 d after transfer. **(C)** Skin was harvested 4 or 8 d after i.d. cell transfer and cultured overnight. Transduced cells were enumerated from the skin and culture supernatant (S/N) and shown as a ratio. **(D–F)** Effector gBT-I or OT-I T cells were transduced with control or S1PR5 RVs and cotransferred i.v. into naive mice whose flanks were treated with DNFB. **(D)** Mice were i.v. labeled before harvest. **(D and E)** Ratios of Ctrl-RV and S1PR5-RV cells were enumerated from the indicated organs and normalized to numbers obtained from the spleen at the indicated times after transfer. **(F)** CD69, CXCR6, and CD103 expression on Ctrl-RV (black) or S1PR5-RV (blue) cells isolated from the indicated organs 8 d after transfer. **(G)** P14 T cells were transduced with control or S1PR5 RVs and cotransferred i.v. into mice infected with LCMV and treated with DNFB. Transduced cells were isolated from the indicated tissues 8 d after transfer. **(H)** Effector CD8^+^ T cells from WT (CD45.1^+^CD45.2^+^) or *Ccr7^−/−^* (CD45.2^+^) were transduced with Ctrl-RV or S1PR5-RV and transferred i.d. into CD45.1 recipient mice. Shown is the ratio *Ccr7^−/−^* and WT cells from the skin 7 d after transfer. In A and B, data are representative of two independent experiments, with *n* = 6–8 mice per experiment. In C, data are pooled from two independent experiments, with *n* = 4 mice per time point. In D, data are representative of two independent experiments, with *n* = 6–8 mice per time point per experiment. In E–G, data are representative of two to four independent experiments, with *n* = 6–10 mice per experiment. In H, data are pooled from three independent experiments, with *n* = 4 mice per group per experiment. Unpaired *t* test was used in A, multiple Wilcoxon test in C, multiple Mann–Whitney test in D, Kruskal-Wallis test in E and G, and Mann–Whitney test in H. *, P < 0.05; ***, P < 0.001; ****, P < 0.0001. SG, salivary gland; T_EFF_, effector T cell.

T_RM_ cells arise from precursor cells recruited to peripheral tissues during acute inflammation (Mackay et al., 2013; Sheridan et al., 2014). G-coupled protein receptors, including CCR7 and S1PR1, can promote egress of these cells to oppose T_RM_ cell generation (Bromley et al., 2013; Mackay et al., 2013; Skon et al., 2013). To determine whether S1PR5 can also facilitate T cell migration from peripheral tissues and preclude T_RM_ cell development, we injected effector CD8^+^ T cells transduced with S1PR5-RV or Ctrl-RV directly into the skin. This approach precipitates local T_RM_ cell formation in the absence of local antigen recognition (Mackay et al., 2013). 8 d after intradermal injection, S1PR5-RV cells were numerically reduced in the skin but not the spleen compared with Ctrl-RV cells, suggesting that sustained S1PR5 expression promotes departure of effector T cells from the skin (Fig. 4, A and B). Corroborating this, culturing explanted skin containing control and S1PR5-RV cells cultured overnight revealed increased emigration of S1PR5-RV cells from the skin tissue (Fig. 4 C). Given our results showing that S1PR5 limited cellular homing to lymphoid organs (Fig. 3 F), we asked whether S1PR5 signals could also inhibit T cell entry into the skin. To address this, we forced S1PR5 expression in effector CD8^+^ T cells that were transferred i.v. into mice treated on the skin flank with the contact sensitizer 1-fluor-2,4-dinitrobenzol (DNFB) to induce T cell migration to this tissue (Frizzell et al., 2020). While equivalent numbers of S1PR5-RV cells were recovered from the spleen, reduced numbers of S1PR5-expressing cells were isolated from the skin as early as 4 d after transfer, a defect that was augmented over time (Fig. 4 D). To extend these observations to other organs, we forced S1PR5 expression in effector CD8^+^ T cells that were transferred i.v. (Fig. 4, E and F) and also examined P14 transgenic CD8^+^ T cells specific for the LCMV glycoprotein (gp_33–41_) that were transferred into LCMV-Armstrong–infected mice (Fig. 4 G). In these settings, T_RM_ cells naturally develop in a wide range of tissues (Casey et al., 2012), and to induce T cell lodgment in the skin, mice were treated with the contact sensitizer DNFB (Frizzell et al., 2020). 8 d after transfer, S1PR5-overexpressing CD69^+^ T cells were comparatively reduced in the liver, salivary glands, small intestine intra-epithelial lymphocytes (SI-IELs), and skin. Furthermore, the expression of T_RM_ cell–associated molecules, including CD69, CXCR6, and CD103, were diminished in S1PR5-overexpressing T cells (Fig. 4 F). This loss of S1PR5-RV cells did not appear linked to increased cell death in the tissue (Fig. S3 D) but likely reflects their relocalization to the vasculature (Figs. 3, B–D; and S3 C). Collectively, these data suggest that irrespective of the mode of T_RM_ cell generation, S1PR5 expression hinders CD8^+^ T_RM_ cell differentiation by dampening T cell extravasation and promoting T cell egress from nonlymphoid tissues.

While these findings showed that S1PR5 expression was sufficient to induce T cell exit from peripheral tissues, it was unclear how S1PR5 might intersect with other molecules coordinating tissue retention. CCR7 promotes memory T cell egress from the skin via lymphatics (Bromley et al., 2013), and CCR7 deficiency enhances skin T_RM_ cell differentiation (Mackay et al., 2013). We therefore asked whether S1PR5 could interface with CCR7 signaling to influence CD8^+^ T cell tissue egress decisions. Consistent with our prior work (Mackay et al., 2013), *Ccr7^−/−^* effector T cells transduced with a Ctrl-RV displayed enhanced retention in the skin after intradermal injection compared with WT T cells (Fig. 4 H). Forced expression of S1PR5 in *Ccr7^−/−^* T cells restored their capacity to egress from the skin (Fig. 4 H), indicating that S1PR5 signaling can override loss of CCR7 to facilitate tissue egress of CD8^+^ T cells.

### TGF-β enforces retention of T_RM_ cell precursors in the skin via *Zeb2* and *S1pr5* downregulation

**Figure 5.**
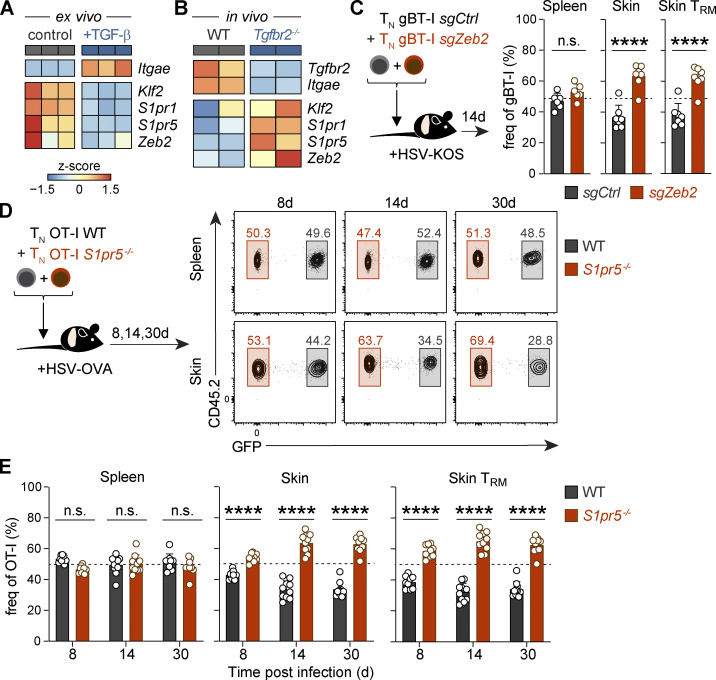
**TGF-β downregulates ZEB2 and enforces the retention of T_RM_ cell precursors in the skin. (A)** Naive P14 CD45.1^+^ T cells were transferred into recipient mice infected with LCMV. P14 T cells were isolated from the spleen 7 dpi and cultured with TGF-β in vitro for 2 d. Expression of the indicated genes was determined by qPCR and represented as a heatmap (*z*-score normalized by row). **(B)** Effector CD45.1^+^CD45.2^+^ OT-I (OT-I WT) and CD45.1^+^ OT-I *Tgfbr2^−/−^* (OT-I *Tgfbr2^−/−^*) were transferred into mice infected with HSV-OVA. OT-I T cells from the skin were isolated 14 dpi, and expression of the indicated genes determined by RNA sequencing and represented as a heatmap (*z*-score normalized by row). **(C)** Naive gBT-I T cells were nucleofected with control-nontargeting (GFP^+^ gBT-I *sgCtrl*) or *Zeb2*-targeting (CD45.1^+^ gBT-I *sgZeb2*) sgRNA/Cas9 RNPs and cotransferred into WT mice. Recipient mice were infected with HSV, and relative frequencies of gBT-I T cells were quantified in the spleen and skin (total gBT-I or CD69^+^CD103^+^ gBT-I T_RM_) 14 dpi. Data are representative of two independent experiments, with *n* = 7–9 mice per experiment. **(D and E)** Naive OT-I T cells sufficient (OT-I WT) or deficient in S1PR5 (OT-I *S1pr5*^−/−^) were transferred at a 1:1 ratio into recipient mice that were subsequently infected with HSV-OVA. Shown are relative frequencies of WT and *S1pr5^−/−^* OT-I T cells isolated from the spleen and skin (total OT-I or CD69^+^CD103^+^ OT-I T_RM_) at 8, 14, and 30 dpi. In C, data are representative of two independent experiments, with *n* = 7–9 mice per experiment. In D and E, data are representative of four independent experiments with *n* = 9–10 mice per group per experiment. Unpaired *t* test was used in C and two-way ANOVA in E. ****, P < 0.0001. freq, frequency; n.s., not significant; T_N_, naive T cell.

**Figure S4.**
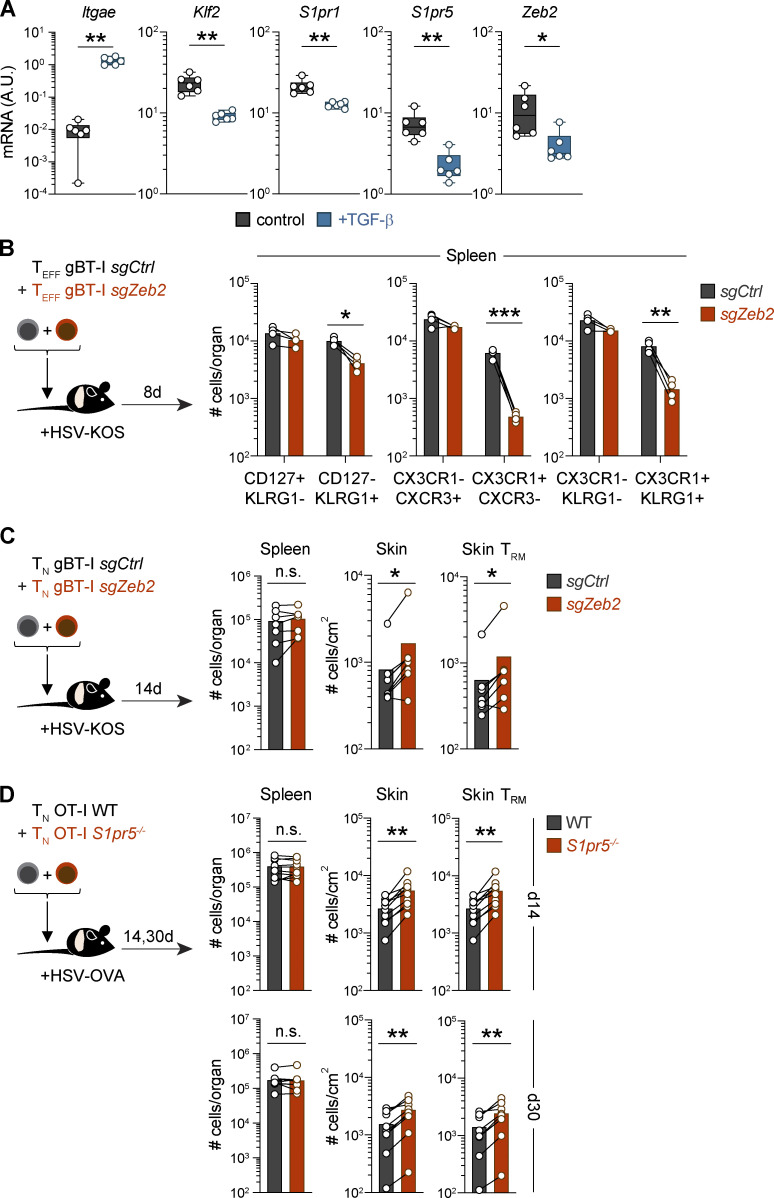
**ZEB2 and S1PR5-deficient CD8^+^ T cells show increased T_RM_ cell formation in the skin. (A)** Naive P14 T cells were transferred into LCMV-infected mice. P14 T cells were isolated from the spleen 7 dpi and cultured with TGF-β in vitro for 2 d. Expression of indicated genes was quantified by qPCR and normalized to a housekeeping gene. **(B and C)** Effector or naive gBT-I T cells were nucleofected with control-nontargeting (GFP^+^ gBT-I *sgCtrl*) or *Zeb2*-targeting (CD45.1^+^ gBT-I *sgZeb2*) sgRNA/Cas9 RNPs and cotransferred into recipient mice infected with HSV. gBT-I T cells were quantified in the spleen (CD127^+^CXCR3^+^KLRG1^−^CX3CR1^−^ and CD127^−^CXCR3^−^KLRG1^+^CX3CR1^+^) 8 dpi (B) or spleen and skin (total gBT-I or CD69^+^CD103^+^ gBT-I) 14 dpi (C). **(D)** Naive OT-I WT and OT-I *S1pr5*^−/−^ T cells were cotransferred into recipient mice infected with HSV-OVA. Shown are numbers of WT and *S1pr5^−/−^* OT-I T cells isolated from the spleen and skin (total OT-I or CD69^+^CD103^+^ OT-I) 14 and 30 dpi. In A, data are pooled from two independent experiments, with *n* = 6 mice per group. In B and C, data are representative of two independent experiments, with *n* = 4 mice (B) and *n* = 7–9 mice (C) per experiment. In D, data are representative of four independent experiments, with *n* = 9–10 mice per group per experiment. Mann–Whitney test was used in A, paired *t* test in B, and paired Wilcoxon test in C and D. *, P < 0.05; **, P < 0.01; ***, P < 0.001. A.U., arbitrary units; n.s., not significant; T_EFF_, effector T cell; T_N_, naive T cell.

Tissue-derived cytokines, including IL-15 and TGF-β, play a crucial role in shaping multiple aspects of T_RM_ cell differentiation (Mackay et al., 2013, 2015b; Schenkel et al., 2016; Zhang and Bevan, 2013). Among these, TGF-β has previously been shown to extinguish expression of *Klf2* and *S1pr1* (Skon et al., 2013), T-bet (Mackay et al., 2015b), and *Zeb2* (Guan et al., 2018). To explore the impact of TGF-β signaling on S1PR5 expression, we cultured effector CD8^+^ T cells with TGF-β in vitro and found that this cytokine drives *S1pr5* downregulation alongside *Klf2*, *S1pr1*, and *Zeb2* (Figs. 5 A and S4 A). To assess the importance of TGF-β signaling in vivo, we transferred effector OT-I T cells that were either sufficient or deficient in the TGF-β receptor II (TGF-βRII; OT-I WT or *Tgfbr2*^−/−^) into HSV-OVA–infected mice. While TGF-βRII–deficient OT-I T cells were compromised in their ability to form T_RM_ cells and almost entirely lacking from the skin, those that were recoverable from the skin epidermis 2 wk after cell transfer exhibited elevated expression of *Klf2* and *S1pr1* compared with OT-I WT T cells. Importantly, TGF-βRII–deficient cells also displayed increased expression of *Zeb2* and *S1pr5*, suggesting that TGF-β signaling might also repress the ZEB2–S1PR5 axis to promote skin T_RM_ cell formation (Fig. 5 B).

Given that ZEB2 can induce *S1pr5* expression (Fig. 2, C and D) and that S1PR5 signaling drives T cell egress from the skin (Fig. 4, A–D), we reasoned that *Zeb2* downregulation via TGF-β signaling may be necessary to promote efficient T_RM_ cell formation. To address whether loss of ZEB2 might enhance skin T_RM_ cell development, we transferred CRISPR/Cas9 *Zeb2*-ablated naive gBT-I T cells to mice that were subsequently infected with HSV. Consistent with previous reports (Dominguez et al., 2015; Omilusik et al., 2015), ZEB2 ablation led to a defect in SLEC differentiation without altering the number of circulating memory precursor cells (Fig. S4 B). In addition, ZEB2 disruption led to increased formation of CD69^+^CD103^+^ T_RM_ cells in the skin 2 wk after HSV infection (Figs. 5 C and S4 C). To assess the relative importance of S1PR5 in this process, we cotransferred congenically distinct OT-I WT T cells (OT-I WT) or *S1pr5*-deficient OT-I T cells (OT-I *S1pr5*^−/−^) into CD45.1 recipient mice before HSV-OVA infection (Fig. 5 D). Akin to ZEB2 ablation, we found that S1PR5 deficiency led to enhanced retention of CD8^+^ T cells in the skin as early as 8 dpi, with an increased potential to form CD69^+^CD103^+^ T_RM_ cells (Fig. 5, D and E; and Fig. S4 D). In contrast, equal proportions of WT and *S1pr5*-deficient CD8^+^ T cells localized to the spleen (Fig. 5, D and E; and Fig. S4 D). Together, our data highlight the importance of TGF-β signaling in downregulating *Zeb2* and *S1pr5* expression, which ultimately enforces the local retention of T cells in peripheral tissues and their differentiation into T_RM_ cells.

### S1PR5 disruption alters the tissue distribution of NK cells and ILC1

**Figure 6.**
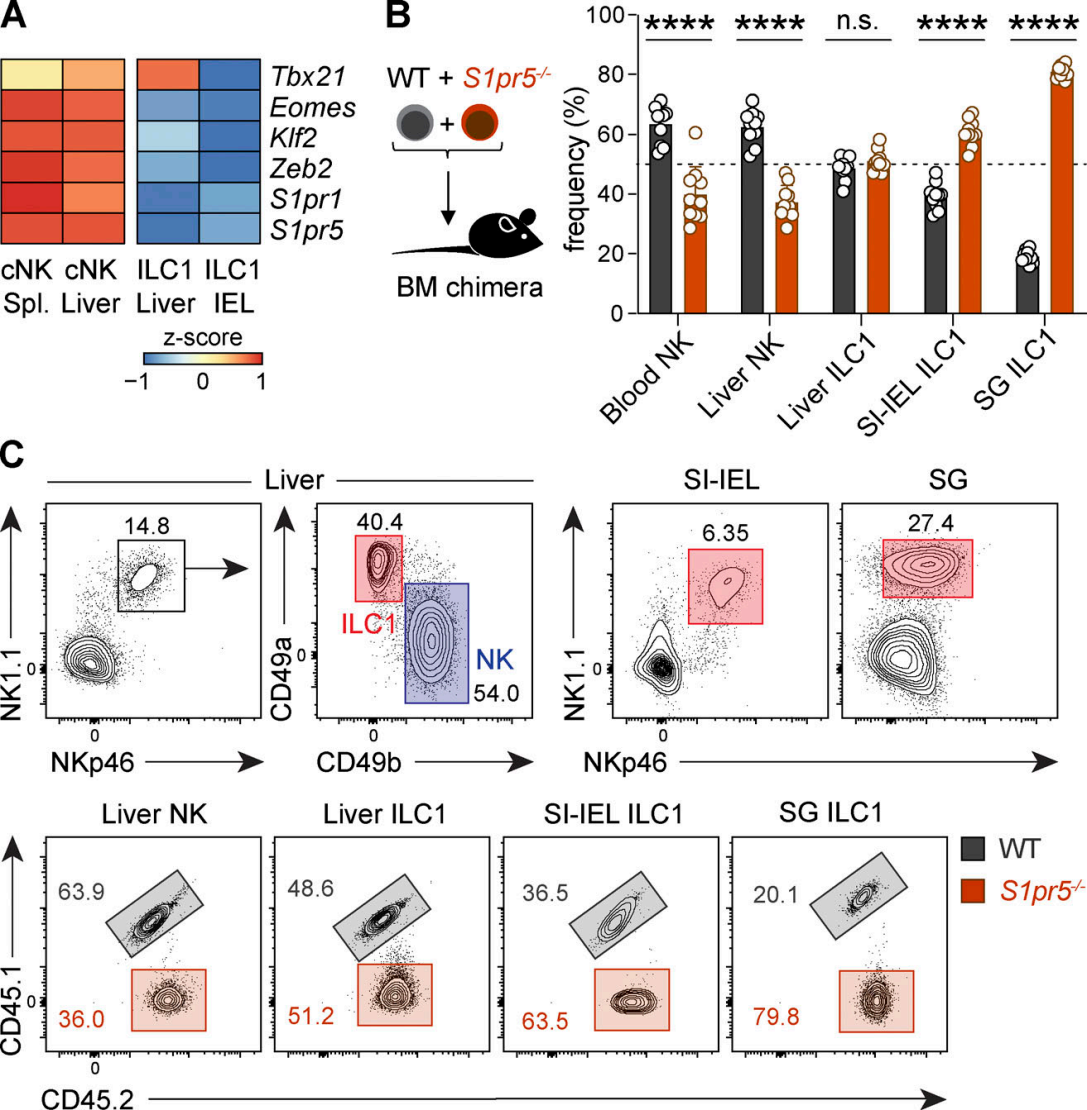
**S1PR5-deficiency results in altered NK and ILC1 tissue distribution. (A)** Heatmap represents of gene expression (*z*-score normalized by row) of mouse NK and ILC1 cells isolated from spleen, liver, and SI-IEL. Gene expression was extracted from the ImmGen Consortium (GEO under accession no. GSE37448). **(B and C)** WT (CD45.1^+^CD45.2^+^) and *S1pr5^−/−^* (CD45.2^+^) BM cells were cotransferred at a 1:1 ratio into lethally irradiated CD45.1 recipient mice. Donor-derived (CD45.2^+^) NK and ILC1 cells were analyzed from indicated organs >8 wk after transfer. Shown are relative frequencies of WT and *S1pr5^−/−^* of NK and ILC1 cells (B) and corresponding gating strategies (C). Data are representative of three independent experiments, with *n* = 9–10 per experiment. ****, P < 0.0001 by two-way ANOVA. n.s., not significant; SG, salivary gland; Spl., spleen.

Tissue residency is not just a feature of CD8^+^ T cells but also a property shared by both innate and adaptive lymphocytes that use conserved mechanisms to differentiate and persist in peripheral tissues (Gasteiger et al., 2015; Mackay et al., 2016; Robinette et al., 2015). While S1PR5 controls the migration of NK cells from the BM and LNs to the peripheral circulation (Jenne et al., 2009; Walzer et al., 2007), the importance of this receptor in regulating the tissue retention of other ILCs is not known. To explore whether the ZEB2–S1PR5 axis may influence the distribution of additional populations of tissue-resident lymphocytes, we compared the expression of various molecules influencing tissue egress in NK cells and tissue-resident ILC1 in publicly available datasets (Fig. 6 A; Robinette et al., 2015). Analogous to CD8^+^ T_RM_ cells, tissue-resident ILC1 displayed lower expression of *Klf2*, *S1pr1*, *S1pr5*, and *Zeb2* compared with their circulating NK cell counterparts (Fig. 6 A). To understand the importance of S1PR5 during ILC1 differentiation in peripheral tissues, we generated mixed BM chimeras by transferring equal proportions of WT (CD45.1^+^CD45.2^+^) and *S1pr5*^−/−^ (CD45.2^+^) cells to CD45.1^+^ hosts. In agreement with prior studies (Jenne et al., 2009; Mayol et al., 2011; Walzer et al., 2007), *S1pr5*^−/−^ NK cells were found in lower frequencies within the blood and liver. In stark contrast, S1PR5 deficiency led to an increased accumulation of ILC1 in the small intestine and salivary glands (Fig. 6, B and C). Taken together, these results argue that S1PR5 commonly regulates the retention of multiple tissue-resident lymphocyte populations across both innate and adaptive cell lineages.

## Discussion

T_RM_ cells are recognized as essential mediators of host protection against cancer and infections in the periphery (Park et al., 2019; Szabo et al., 2019), yet the processes regulating their generation are not fully understood. One of the key events controlling T_RM_ cell commitment is the downregulation of molecules that promote tissue exit. In the current study, we identify the ZEB2–S1PR5 axis as a novel pathway balancing peripheral T cell trafficking and T_RM_ cell formation, with S1PR5 downregulation being required for efficient T_RM_ cell differentiation. We find that while S1PR5 has a complementary function to S1PR1, these two receptors display distinct expression patterns and transcriptional regulation. Despite this, local cues, such as TGF-β, act in an overarching manner to suppress both S1PR1 and S1PR5 expression and in doing so, coordinate tissue retention. As a consequence, while multiple independent molecular mechanisms converge to control T cell tissue exit, there appears to exist a unifying process that harmonizes these pathways and enforces T_RM_ cell commitment.

It is known that S1P signaling regulates T cell migration and positioning in nonlymphoid organs (Baeyens and Schwab, 2020). We have previously shown that the downregulation of S1PR1 and S1PR5, but not other S1PRs is a conserved feature of the T_RM_ cell differentiation program across distinct tissues (Mackay et al., 2013). Seminal work by Skon et al. (2013) showed that the loss of *S1pr1* is required for CD8^+^ T_RM_ cell development and further, that the forced expression of *S1pr1* precludes T_RM_ cell formation. Here, we extend this observation by showing that in addition to *S1pr1*, *S1pr5* is selectively downregulated in T_RM_ cells and similarly required for T_RM_ cell formation. More importantly, we identified key differences between these receptors with regard to their transcriptional regulation, expression patterns, and impact on T cell trafficking over the course of CD8^+^ T cell differentiation. While *S1pr1* expression is primarily regulated by KLF2 (Carlson et al., 2006), the loss of KLF2 had a negligible effect on *S1pr5* expression. Instead, we observed that the induction of ZEB2 downstream of T-bet is necessary to promote *S1pr5* expression. Interestingly, the sole expression of T-bet does not seem sufficient to induce *S1pr5* since T-bet–expressing lymphocytes, including CD4^+^ and TCRγδ T cells, typically show negligible *S1pr5* mRNA levels. This is likely because T-bet acts as a gradient (Dominguez et al., 2015; Joshi et al., 2007) whereby only high concentrations of T-bet can induce ZEB2 and, consequently, S1PR5. Together, this differential molecular regulation explains discordant S1PR1 and S1PR5 expression in naive CD8^+^ T cells, which express KLF2 (Carlson et al., 2006) but not ZEB2 (Dominguez et al., 2015; Omilusik et al., 2015). Importantly, while S1PR1 primarily acts by promoting T cell egress from peripheral tissues (Skon et al., 2013), we find that S1PR5 additionally affects T cells by hindering tissue infiltration and promoting the exit of interstitial T cells. Given that S1PR5 expression is limited to specific T cell subsets, S1PR5-controlled migration is likely restricted to effector and memory CD8^+^ T cells and may explain why ZEB2-dependant terminal effector T cells, which show the highest S1PR5 expression, are mostly confined to the vasculature and poorly infiltrate peripheral organs (Gerlach et al., 2016). Consequently, targeting S1PR5 or molecular components that induce this molecule may permit more selective manipulation of antigen-experienced T cells without impacting naive T cell trafficking.

After entry to peripheral tissues, effector T cells receive extrinsic cues that either promote their local retention or oblige their return to the circulation. While chemokine gradients can entice migration of T_RM_ cell precursors toward epithelial surfaces (Mackay et al., 2013; Masopust et al., 2010; Takamura et al., 2019), other pathways, including CCR7, S1PR1, and S1PR5 signaling, actively drive their egress via lymphatics (Bromley et al., 2005; Debes et al., 2005; Ledgerwood et al., 2008; Skon et al., 2013). However, the integration of these signals and their impact on the decision to become tissue resident likely depend on the inflammatory context, tissue location, and cell type. For example, although S1PR1 degradation via CD69 complexing is required to enforce the local retention of CD8^+^ T cells in the skin, kidney, and lung (Mackay et al., 2015a; Takamura et al., 2016; Walsh et al., 2019), CD69 expression is dispensable for T cell retention in other tissues, such as the small intestine (Walsh et al., 2019). Differences observed in distinct tissue sites suggest a certain degree of redundancy with regard to egress mechanisms, which could be orchestrated by local cues from the microenvironment. Interestingly, the cytokines IL-15 and TGF-β, which drive keys aspects of T_RM_ cell differentiation, survival, and function (Mackay et al., 2015b; Schenkel et al., 2016; Zhang and Bevan, 2013), are also essential to enforce the downregulation of tissue egress molecules. For instance, IL-15 induces the expression of Hobit, which in turn represses *Ccr7* and *S1pr1* (Mackay et al., 2016). Here, we found that TGF-β promotes the downregulation of both *Klf2* and *Zeb2*, thereby suppressing *S1pr1* and *S1pr5*, respectively, in the skin. While not all T_RM_ cells rely on TGF-β for their development, it is unclear whether such TGF-β–independent T_RM_ cells show a less stable form of tissue residency or might rely more stringently on other retention mechanisms, such as integrins (Christo et al., 2021; McNamara et al., 2017).

The shutdown of tissue egress signals is a key step toward the formation of T_RM_ cells, yet it is not a feature unique to T cells. For instance, the downregulation of egress receptors, such as S1PR1, is widespread among innate lymphocyte populations isolated from peripheral tissues, including NK T cells, mucosal-associated invariant T cells, and ILCs (Huang et al., 2018; Mackay et al., 2016; Salou et al., 2019). Downregulation of tissue egress genes can be enforced via common mechanisms in innate and adaptive lymphocytes, with the transcription factors Blimp1 and Hobit directly repressing *Ccr7* and *S1pr1* in cells spanning both lineages (Mackay et al., 2016). Here, we found that extinguishment of *S1pr5* is an additional conserved program acting to promote tissue residency in both ILCs and CD8^+^ T cells.

While tissue-resident cells provide critical protection against infections and malignancies, they can also be the driving cause of immune pathologies (Park and Kupper, 2015; Sasson et al., 2020). In such settings, therapeutic intervention aiming to selectively remove these deleterious T cells could be highly beneficial but may prove challenging given the durability of T_RM_ cell populations in some tissues. For instance, skin T_RM_ cells are numerically stable and not displaced over time (Park et al., 2018); furthermore, they are inaccessible to targeted depletion using monoclonal antibodies in some circumstances (Watanabe et al., 2015). Therefore, strategies aiming to remove T_RM_ cells by promoting tissue exit via activation of the KLF2–S1PR1 or ZEB2–S1PR5 pathways may help to overcome these obstacles. On the other hand, while skin T_RM_ cells are highly durable, those that reside in the lung decay over time (Slütter et al., 2017). Interestingly, lung CD8^+^ T_RM_ cell attrition can be attributed in part to “retrograde migration” whereby long-term tissue-resident cells relocate from the periphery to the circulation at steady state, but the mechanisms facilitating this process are unknown (Stolley et al., 2020). It is possible that tissue egress gene pathways may be specifically reactivated in certain populations of T_RM_ cells, such as those lacking expression of Id3 (Kurd et al., 2020; Milner et al., 2020). Interestingly, it has recently been shown that compared with Id3^+^ T_RM_ cells, the Id3^−^ population can progressively reexpress ZEB2 and appears to contract within the SI-IEL over time (Milner et al., 2020), potentially via an ZEB2–S1PR5 emigration axis. Elucidating the role of S1PRs in coordinating this process may provide new avenues to promote long-term local immunity for vaccination against mucosal pathogens. Furthermore, S1PR5 may serve as an exit strategy for T_RM_ cells shown to reenter the circulation upon antigen restimulation (Behr et al., 2020; Fonseca et al., 2020; Stolley et al., 2020). Whether antigen recognition may lead to the induction of *Zeb2* and *S1pr5* in T_RM_ cells to facilitate retrograde migration remains an open question.

**Figure S5.**
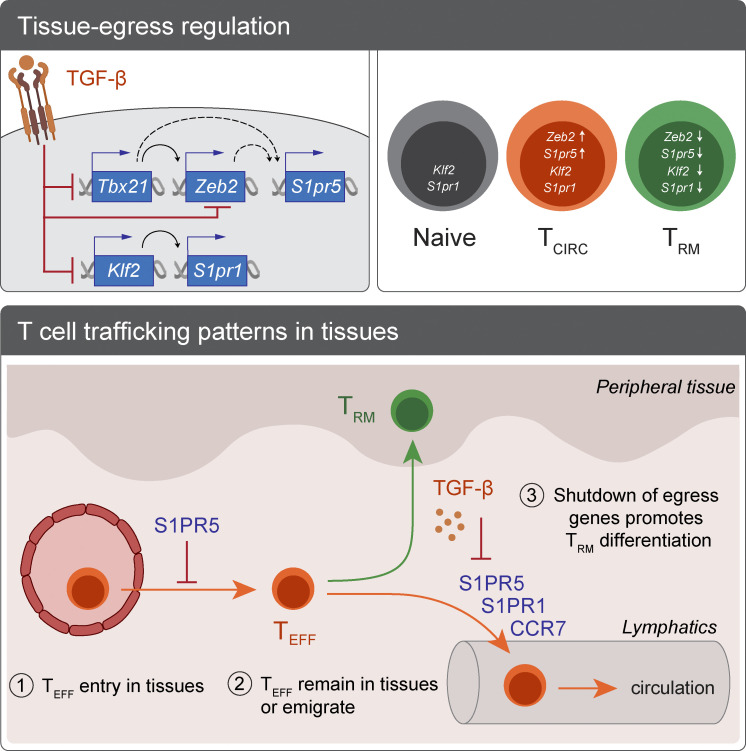
**Model of T cell trafficking in peripheral tissues impacting the generation of T_RM_ cells.** Multiple factors can influence the decision of effector T cells (T_EFF_) to enter inflamed tissues, return to the circulation, or remain locally and differentiate into T_RM_ cells. Local T cell retention is classically achieved via the downregulation of tissue egress molecules, including CCR7 and S1PRs. In this study, we identified S1PR5 as an additional regulator of these decisions, with S1PR5 expression limiting T cell extravasation and promoting T cell egress, thereby impeding T_RM_ cell development. While S1PR1 and S1PR5 share the same ligand, these two receptors are controlled by distinct transcriptional regulators, with KLF2 and ZEB2 being the main drivers of S1PR1 and S1PR5, respectively. Importantly, despite exhibiting distinct transcriptional regulation, the tissue-derived cytokine TGF-β promotes downregulation of both pathways and ultimately *S1pr1* and *S1pr5*, thereby enforcing tissue retention and T_RM_ cell differentiation. T_CIRC_, circulating T cell.

Overall, we reveal that S1PR5 acts as a novel checkpoint that controls bidirectional lymphocyte tissue trafficking and impacts the formation of tissue-resident cells across both innate and adaptive cell lineages. Importantly, we demonstrate that tissue T cell retention is a highly coordinated process dictated by the integration of cytokine cues from the local microenvironment, which unanimously enforce the shutdown of multiple tissue egress genes, even when they are induced by distinct transcriptional regulators. Together, these signals enforce the establishment of tissue residency and promote T_RM_ cell formation (Fig. S5). Further studies will be required to determine how S1PR5 interfaces or combines with other tissue egress factors to control peripheral lymphocyte migration and differentiation. Understanding these interactions will unveil how these pathways could be harnessed for therapeutic gain, such as to improve T_RM_ cell formation during vaccination or elicit the departure of malevolent tissue-resident lymphocytes in the context of autoimmunity.

## Materials and methods

### Mice

C57BL/6, B6.SJL-PtprcaPep3b/BoyJ (CD45.1), B6.SJL-PtprcaPep3b/BoyJ × C57BL/6 (CD45.1 × CD45.2), gBT-I CD45.1, gBT-I GFP, P14 CD45.1, P14 Thy1.1, OT-I CD45.1, OT-I GFP, OT-I *Tbx21^−/−^* CD45.1, OT-I Tgfbr2^f/f^.dLck-cre CD45.1 (OT-I *Tgfbr2^−/−^*), OT-I *S1pr5^−/−^*, and *S1pr5^−/−^* mice were bred in the Department of Microbiology and Immunology at The University of Melbourne. Female mice were used for experiments at 6–12 wk of age. All animal experiments were approved by the University of Melbourne Animal Ethics Committee. *S1pr5^−/−^* mice were kindly provided by J. Chun. P14 mice express a transgenic TCR recognizing the LCMV glycoprotein-derived epitope gp_33–41_. gBT-I mice express a transgenic TCR recognizing the HSV glycoprotein-B–derived epitope gB_498–505_. OT-I mice express a transgenic TCR recognizing the OVA epitope OVA_257–264_. BM chimera were generated by irradiation of recipient mice (550 rad 3 h apart × 2) followed by reconstitution with 2–5 × 10^6^ donor BM cells. Residual lymphocytes were depleted the next day (100 μg anti-Thy1 [T24] i.p.).

### T cell transfer

Adoptive transfers of naive gBT-I, P14, or OT-I T cells were performed i.v. with LN suspensions. Naive gBT-I, P14, or OT-I T cells were transferred at a total number of 5 × 10^4^ or 2.5 × 10^4^ cells/population in cotransfer experiments, where cell types were transferred at a ratio of 1:1.

### Infections and DNFB treatment

HSV infection was performed by scarification using 1 × 10^6^ PFU of the KOS strain of the virus (HSV-KOS) or the KOS strain modified to express OVA protein (HSV-OVA). LCMV infection was performed by i.p. injection of 2 × 10^5^ PFU of the Armstrong strain of LCMV. For DNFB treatment, mice were shaved and depilated before application of 15–20 μl DNFB (0.25%) in acetone and oil (4:1) to a 1.5-cm^2^ region of skin 3 d after LCMV infection.

### Mouse tissue processing

Mice were i.v. injected with 4 μg biotin-conjugated anti-CD45 (30-F11) or CD8β (YTS156.7.7) 4 min before euthanasia, as indicated. Blood was collected via an incision in the submandibular region and then lysed using 1× RBC lysis buffer (eBioscience). Spleens and LNs were processed into a single-cell suspension using metal meshes. Femurs were flushed using a 23-gauge syringe filled with 1× PBS to obtain BM single-cell suspension. Livers were meshed through 70-μm cell strainers, and pellets were resuspended in 35% isotonic Percoll (GE Healthcare) before density gradient centrifugation (500 *g*, 20 min). BM, spleen, and liver RBCs were lysed using 1× RBC lysis buffer (eBioscience). Flank skin was shaved and depilated, and an area of 1–3 cm^2^ was excised. Skin was incubated in Dispase II (2.5 mg/ml; Roche) for 90 min at 37°C. Epidermal and dermal layers were separated, placed in collagenase III (3 mg/ml; Worthington) and DNase I (5 μg/ml; Roche), chopped into fine pieces, and further incubated for 30 min at 37°C. Digested skin was homogenized into a single-cell suspension and sequentially passed through 70 μm and 30 μm nylon mesh. Small intestine was cleared of luminal contents, and Peyer’s patches were excised. Intestines were longitudinally opened and cut into ∼1-cm fragments, which were incubated at 37°C for 30 min with lateral rotation (230 rpm) in 10% HBSS/Hepes containing dithioerythritol (0.15 mg/ml; Sigma-Aldrich). Intraepithelial lymphocytes were then purified using 44/70% Percoll gradient centrifugation. Kidneys and salivary glands were collected in collagenase III (3 mg/ml) and DNase I (5 μg/ml; Roche), chopped into fine pieces, and incubated for 30 min at 37°C. Digested pieces were homogenized and passed through a 70-μm cell strainer, and lymphocytes were purified using 44/70% Percoll gradient centrifugation.

### Human tissue processing

Human peripheral blood and healthy skin were obtained with informed written consent from patients undergoing abdominoplastic surgery. This work was approved by the Ballarat Health Services and St. John of God Hospital Human Research Ethics Committee (ethics number HREC/15/BHSSJOG/5). Peripheral blood mononuclear cells (PBMCs) were prepared by Ficoll gradient centrifugation. T cells were isolated from the skin as previously described (Trubiano et al., 2020). Briefly, skin was incubated in Dispase II solution (2.5 mg/ml; Roche) overnight at 4°C. Epidermal and dermal layers were separated, placed in collagenase III (3 mg/ml; Worthington) and DNase I (5 μg/ml; Roche), chopped into fine pieces, and further incubated for 90 min at 37°C. Skin was homogenized into a single-cell suspension and filtered using 30-μm nylon meshes.

### Flow cytometry and cell sorting

Mouse cells were stained at 4°C for 60 min with the following antibodies (all purchased from BD Biosciences, BioLegend, or Thermo Fisher Scientific): anti-B220 (RA3-6B2), anti-CD8α (53-6.7), anti-CD44 (IM7), anti-CD45.1 (A20), anti-CD45.2 (104), anti-CD49a (Ha31/8), anti-CD49b (DX5), anti-CD62L (MEL-14), anti-CD69 (H1.2F3), anti-CD103 (2E7), anti-CD127 (A7R34), anti-CXCR3 (CXCR3-173), anti-CXCR6 (SA051D1), anti-CX3CR1 (SA011F11), anti-KLRG1 (2F1), anti-Ly6C (HK1.4), anti-NK1.1 (PK136), anti-NKp46 (29A1.4), anti-TCRβ (H57-597), anti-TCRγδ (GL3), and anti-Vα2 (B20.1). PE-conjugated annexin V was purchased from BioLegend, and staining was performed according to the manufacturer’s instructions. In some experiments, cells were fixed and permeabilized using a FoxP3 transcription factor staining buffer set and stained with the following antibodies (purchased from BD Biosciences or Cell Signaling Technology): anti-Bcl2 (3F11) and anti-Bim (C34C5). Human cells were stained with the following antibodies (all purchased from BD Biosciences or BioLegend): anti-CD3 (UCHT1), anti-CD8α (SK1), anti-CD45RO (UCHL1), anti-CD69 (FN5D), and anti-CD103 (Ber-ACT8). Dead cells were excluded from analysis using DAPI (0.5 μM; BioLegend), Zombie Yellow, or Zombie NIR fixable live/dead (BioLegend). For flow cytometry experiments, samples were acquired on a five-laser BD LSRFortessa (BD Biosciences) or a five-laser Cytek Aurora analyzer. For cell sorting experiments, murine T cells from the spleen (CD8a^+^Vα2^+^CD45.1^+/−^) and skin (TCRβ^+^Vα2^+^CD45.1^+/−^) or human T cells from PBMCs (CD3^+^CD8α^+^CD45RO^+^) and skin (CD3^+^CD8α^+^CD45RO^+^CD69^+^CD103^−^ and CD3^+^CD8α^+^CD45RO^+^CD69^+^CD103^+^) were sorted using a five-laser BD FACSAria III (BD Biosciences; >95% purity). Data were analyzed using FlowJo version 10 (TreeStar).

### RV transduction of CD8^+^ T cells

RVs were produced using Plat-E cells (Cell Biolabs), which were transfected with pCL-Eco and pMSCV-IRES-GFP II (pMIG II)– or pMSCV-IRES-Thy1.1–based vectors. Briefly, Plat-E cells were seeded in 96-mm dishes at a density of 7 × 10^6^ cells 12 h before transfection with 14 μg of pMIG II and 7 μg of pCL-Eco plasmid DNA using the CalPhos Mammalian Transfection Kit (Takara). Viral supernatants were harvested 48 h later and filtered (0.45 μm; Millipore). T-bet and KLF2 vectors have been previously described (Mackay et al., 2015b; Skon et al., 2013). *S1pr5* and *Zeb2* cDNA were cloned into pMIG II vector. Purified naive gBT-I, P14, or OT-I CD8^+^ T cells were in vitro activated with anti-CD3 (145-2C11) and anti-CD28 (37.51, 5 μg/ml for each; both from Bio X Cell) for 24 h and were “spinfected” with 0.5 ml of retroviral supernatant in 24-well plates coated with Retronectin (32 μg/ml; Takara). CD8^+^ T cells were further expanded for 3 d in the presence of IL-2 (25 U/ml; Peprotech). Transduction efficiency was determined by GFP expression. Cells transduced with an empty vector (Ctrl-RV) or overexpression vectors (Tbet-RV or S1PR5-RV) were mixed at a 1:1 ratio. 2 × 10^5^ transduced cells of the relevant specificity were administered i.v. in mice that were infected with HSV 2 d before or LCMV 1 d before. In the absence of infection, 2–5 × 10^6^ transduced cells were i.v. or intradermally injected into naive recipients. In some experiments, cells transduced with an empty vector (Ctrl-RV) or overexpression vector (ZEB2-RV) were maintained in culture for 3 d in the presence of IL-15 (10 ng/ml; Peprotech), and GFP^+^ cells were sorted by flow cytometry before quantification of gene expression using quantitative PCR (qPCR).

### Skin explant migration assay

Flank skin was shaved and depilated, and an area of 1 cm^2^ was excised. Skin samples were cultured overnight in complete RPMI medium. The next day, cells that migrated out of the explant were collected from the culture supernatant, whereas cells that did not migrated from the skin tissue were isolated as indicated above. After processing, cells were enumerated by flow cytometry.

### T cell culture with cytokines

Mice received naive P14 T cells and were infected with LCMV as described above. 7 dpi, P14 T cells were sorted from the spleen (CD8a^+^Vα2^+^CD45.1^+^) and cultured in the presence of IL-15 (10 ng/ml) and TGF-β (10 ng/ml) for 48 h before quantification of gene expression by qPCR.

### CRISPR/Cas9 gene editing of CD8^+^ T cells

Single guide RNA (sgRNA) targeting *Cd19* (5′-CCU​GGC​CUG​GGA​UUG​CAC​GU-3′ and 5′-GAG​AAG​CUG​GCU​UGG​UAU​CG-3′), *Klf2* (5′-CUG​GCC​GCG​AAA​UGA​ACC​CG-3′ and 5′-UCC​AUG​GGA​UUG​GAC​GGU​CU-3′), *Tbx21* (5′-GGC​AGU​CAC​UGC​AAU​GAA​CU-3′ and 5′-GGU​ACU​UGU​GGA​GAG​ACU​GC-3′), *Zeb2* (5′-ACU​ACU​GGA​AGA​CCG​ACA​GG-3′ and 5′-CAG​AGU​CCA​AUG​CAG​CAC​UU-3′), or scramble control (5′-GCA​CUA​CCA​GAG​CUA​ACU​CA-3′) were purchased from Synthego (CRISPRevolution sgRNA EZ Kit). sgRNA/Cas9 RNPs were formed by incubating 1 μl sgRNA (0.3 nmol/μl) with 0.6 μl Alt-R S.p. Cas9 Nuclease V3 (10 mg/ml; Integrated DNA Technologies) for 10 min at room temperature. Naive or in vitro–activated (anti-CD3 and anti-CD28, 5 μg/ml each for 24–48 h) gBT-I T cells were resuspended in 20 μl P3 buffer (P3 Primary Cell 4D-Nucleofector X Kit S; Lonza), mixed with sgRNA/Cas9 RNP, and electroporated using a Lonza 4D-Nucleofector system (pulse code: DN100 for naive T cells and CM137 for activated T cells). Naive gBT-I T cells were rested for 10 min at 37°C before i.v. transfer, whereas activated gBT-I T cells were further expanded for 3 d in the presence of IL-2 (25 U/ml; Peprotech). 2 × 10^5^ gBT-I T cells edited with control (*sgCtrl*) and *Zeb2* (*sgZeb2*) guides were mixed at a 1:1 ratio and transferred i.v. Mice receiving edited naive T cells were infected with HSV 4 d after cell transfer, whereas mice that received edited in vitro–activated cells were infected with HSV 2 d before transfer. In some experiments, in vitro–activated P14 T cells were electroporated as mentioned above with control *Cd19* (sgCd19) or *Klf2* (sgKlf2) targeting guides in P4 buffer (P4 Primary Cell 4D-Nucleofector X Kit S). Cells were further maintained in culture for 3 d in the presence of IL-15 (10 ng/ml; Peprotech) and then performed quantification of gene expression using qPCR. In other experiments, in vitro activated P14 T cells were transduced as mentioned above with pMSCV-IRES-Thy1.1 empty vector (Ctrl-RV) or KLF2 overexpression vectors (KLF2-RV) and were subsequently nucleofected 2 d later with *Cd19* (*sgCd19*) or *Tbx21* (*sgTbx21*) targeting guides in P4 buffer (P4 Primary Cell 4D-Nucleofector X Kit S; Lonza). Cells were further maintained in culture for 3 d in the presence of rhIL-2 (100 U/ml, Peprotech), and Thy1.1^+^ cells were sorted by flow cytometry before quantification of gene expression using qPCR.

### qPCR

RNA was extracted from sorted samples using RNeasy Plus Micro Kit (QIAGEN) according to the manufacturer’s instructions. mRNA was converted into cDNA using High Capacity cDNA Reverse Transcription Kit or SuperScript IV VILO Master Mix (Thermo Fisher Scientific), and genes of interest were preamplified using TaqMan PreAmp Master Mix (Thermo Fisher Scientific). Gene expression was analyzed by real-time PCR using the StepOnePlus Real-Time PCR System (Thermo Fisher Scientific), TaqMan Fast Advanced Master Mix, and the following TaqMan probes (purchased from Thermo Fisher Scientific or Integrated DNA Technologies): mouse probes, *Hprt* Mm00446968_m1, *Klf2* Mm01244979_g1, *S1pr1* Mm00514644_m1, *Tbp* Mm00446973_m1, *Tbx21* Mm00450960_m1, and *Zeb2* Mm.PT.58.7239300. *S1pr5* probe was custom made using the following primers: forward primer 5′-ACC​AAG​ACT​CCT​CCA​ACA-3′, TaqMan probe 5′-AAC​CTT​GGA​TCG​CAG​CTC​TAG​CC-3′, reverse primer 5′-GGG​AGA​CAA​GTG​TTC​TGA​TG-3′; and human probes, *KLF2* Hs00360439*_*g1, *S1PR1* Hs00173499*_*m1, *S1PR5* Hs00928195_s1, *UBC* Hs00824723_m1. In some experiments, gene expression was assessed with an ABI 7000 sequence-detection system, and amplification was detected with PowerUp SYBR Green Master Mix (Applied Biosystems). The sequences of the primer pairs used were as follows: *Klf2*, forward 5′-ACC​AAC​TGC​GGC​AAG​ACC​TA-3′ and reverse 5′-CAT​CCT​TCC​CAG​TTG​CAA​TGA-3′; *S1pr1*, forward 5′-GTG​TAG​ACC​CAG​AGT​CCT​GCG-3′ and reverse 5′-AGC​TTT​TCC​TTG​GCT​GGA​GAG-3′; *S1pr5*, forward 5′-GCC​TGG​TGC​CTA​CTG​CTA​CAG-3′ and reverse 5′-CCT​CCG​TCG​CTG​GCT​ATT​TCC-3′; *Tbx21*, forward 5′-CAA​CAA​CCC​CTT​TGC​CAA​AG-3′ and reverse 5′-TCC​CCC​AAG​CAG​TTG​ACA​GT-3′; *Zeb2*, forward 5′-CAT​GAA​CCC​ATT​TAG​TGC​CA-3′ and reverse 5′-AGC​AAG​TCT​CCC​TGA​AAT​CC-3′ or forward 5′-CCA​GAG​GAA​ACA​AGG​ATT​TCA​G-3′ and reverse 5′-AGG​CCT​GAC​ATG​TAG​TCT​TGT​G-3′; and *Hprt*, forward 5′-CAT​TAT​GCC​GAG​GAT​TTG​GAA-3′ and reverse 5′-CAC​ACA​GAG​GGC​CAC​AAT​GT-3′. Cycle-threshold values were determined for genes individually, and gene expression was normalized according to the 2^-dCt^ method to the housekeeping gene *Hprt* or *Tbp* for mouse samples or *UBC* for human samples.

### RNA sequencing

cDNA library preparation, sequencing, normalization of sequenced reads, and analysis of differentially expressed genes have been described elsewhere (Christo et al., 2021). Heatmap representation of selected differentially expressed genes was generated using *pheatmap*. RNA sequencing data have been deposited at the National Center for Biotechnology Information Gene Expression Omnibus (GEO) public database under accession no. GSE178768.

### Confocal microscopy and image analysis

Spleens were prepared for immunofluorescence staining as described previously (Kato et al., 2015). Briefly, spleens were fixed for 8 h at 4°C in PLP buffer (0.2 M NaH_2_PO_4_, 0.2 M Na_2_HPO_4_, 0.2 M L-lysine, and 0.1 M sodium periodate with 2% paraformaldehyde), washed twice in PBS, embedded in optimal cutting temperature compound (Tissue Tek; Sakura Finetek), snap frozen in liquid nitrogen, and stored at −80°C. Frozen tissues were sectioned to 12-μm thickness with a cryostat (CM3050S; Leica). Sections were fixed with acetone, blocked for 10 min with Serum-Free Protein Block (Dako), and stained with the following antibodies (all from BioLegend): anti-B220 (RA3-6B2) Pacific Blue, anti-GFP (FM264G) AF488, and anti-CD31 (390) AF647. Slides were mounted using ProLong Gold Antifade Reagent (Invitrogen). Mosaic imaging covering ∼1,600 × 3,700 μm were acquired with a 20× objective for each spleen on an LSM 700 confocal microscope. Images were processed using Imaris (Bitplane). Delineation of spleen WP and RP and quantification of retrovirally transduced GFP^+^ cells in these areas were performed semiautomatically using the Imaris “surface” tool.

### Statistical analysis

All statistics analyses were calculated by performing unpaired *t* test, unpaired Mann–Whitney test, paired Wilcoxon test, one-way ANOVA test with Dunnet posttest, or two-way ANOVA test with Šidák posttest using Prism 8 (GraphPad Software). *, P < 0.05; **, P < 0.01; ***, P < 0.001; and ****, P < 0.0001.

### Online supplemental material

Fig. S1 shows the effect of KLF2 ablation or overexpression on the expression of *Klf2*,* S1pr1*,* S1pr5*,* Tbx21*, and *Zeb2* in effector CD8^+^ T cells. Fig. S2 shows the effect of T-bet or ZEB2 ablation or overexpression on the expression of *Klf2*,* S1pr1*,* S1pr5*,* Tbx21*, and *Zeb2* and SLEC differentiation in effector CD8^+^ T cells in various settings. Fig. S3 depicts the degree of *S1pr5* overexpression in S1PR5-transduced cells and its effect on their vascular localization and regulation of apoptosis. Fig. S4 illustrates the impact of TGF-β signaling on the expression of various genes, including *S1pr5*, and shows the effect of *Zeb2* and *S1pr5* ablation of CD8^+^ T cell differentiation. Fig. S5 shows a model of how S1PR5 expression is regulated in CD8^+^ T cells and how it affects their trafficking and differentiation into T_RM_ cells.

## References

[bib1] Anderson, K.G., K. Mayer-Barber, H. Sung, L. Beura, B.R. James, J.J. Taylor, L. Qunaj, T.S. Griffith, V. Vezys, D.L. Barber, and D. Masopust. 2014. Intravascular staining for discrimination of vascular and tissue leukocytes. Nat. Protoc. 9:209–222. 10.1038/nprot.2014.005 24385150 PMC4428344

[bib2] Baeyens, A.A.L., and S.R. Schwab. 2020. Finding a way out: S1P signaling and immune cell migration. Annu. Rev. Immunol. 38:759–784. 10.1146/annurev-immunol-081519-083952 32340572

[bib3] Behr, F.M., L. Parga-Vidal, N.A.M. Kragten, T.J.P. van Dam, T.H. Wesselink, B.S. Sheridan, R. Arens, R.A.W. van Lier, R. Stark, and K. van Gisbergen. 2020. Tissue-resident memory CD8(+) T cells shape local and systemic secondary T cell responses*. *Nat*.* Immunol. 21:1070–1081. 32661361 10.1038/s41590-020-0723-4

[bib4] Bromley, S.K., S.Y. Thomas, and A.D. Luster. 2005. Chemokine receptor CCR7 guides T cell exit from peripheral tissues and entry into afferent lymphatics. Nat. Immunol. 6:895–901. 10.1038/ni1240 16116469

[bib5] Bromley, S.K., S. Yan, M. Tomura, O. Kanagawa, and A.D. Luster. 2013. Recirculating memory T cells are a unique subset of CD4+ T cells with a distinct phenotype and migratory pattern. J. Immunol. 190:970–976. 10.4049/jimmunol.1202805 23255361 PMC3618989

[bib6] Carlson, C.M., B.T. Endrizzi, J. Wu, X. Ding, M.A. Weinreich, E.R. Walsh, M.A. Wani, J.B. Lingrel, K.A. Hogquist, and S.C. Jameson. 2006. Kruppel-like factor 2 regulates thymocyte and T-cell migration. Nature. 442:299–302. 10.1038/nature04882 16855590

[bib61] Casey, K.A., K.A. Fraser, J.M. Schenkel, A. Moran, M.C. Abt, L.K. Beura, P.J. Lucas, D. Artis, E.J. Wherry, K. Hogquist, . 2012. Antigen-independent differentiation and maintenance of effector-like resident memory T cells in tissues. J. Immunol. 188:4866–4875. 10.4049/jimmunol.1200402 22504644 PMC3345065

[bib7] Christo, S.N., and M. Evrard, S.L. Park, L.C. Gandolfo, T.N. Burn, R. Fonseca, D.M. Newman, Y.O. Alexandre, N. Collins, N. Zamudio, . 2021. Discrete tissue microenvironments instruct diversity in resident memory T cell function and plasticity. Nat. Immunol. 22: 1140–1151. 34426691 10.1038/s41590-021-01004-1

[bib8] Debes, G.F., C.N. Arnold, A.J. Young, S. Krautwald, M. Lipp, J.B. Hay, and E.C. Butcher. 2005. Chemokine receptor CCR7 required for T lymphocyte exit from peripheral tissues. Nat. Immunol. 6:889–894. 10.1038/ni1238 16116468 PMC2144916

[bib9] Dominguez, C.X., R.A. Amezquita, T. Guan, H.D. Marshall, N.S. Joshi, S.H. Kleinstein, and S.M. Kaech. 2015. The transcription factors ZEB2 and T-bet cooperate to program cytotoxic T cell terminal differentiation in response to LCMV viral infection. J. Exp. Med. 212:2041–2056. 10.1084/jem.20150186 26503446 PMC4647261

[bib10] Fonseca, R., L.K. Beura, C.F. Quarnstrom, H.E. Ghoneim, Y. Fan, C.C. Zebley, M.C. Scott, N.J. Fares-Frederickson, S. Wijeyesinghe, E.A. Thompson, . 2020. Developmental plasticity allows outside-in immune responses by resident memory T cells. Nat. Immunol. 21:412–421. 10.1038/s41590-020-0607-7 32066954 PMC7096285

[bib11] Frizzell, H., R. Fonseca, S.N. Christo, M. Evrard, S. Cruz-Gomez, N.G. Zanluqui, B. von Scheidt, D. Freestone, S.L. Park, H.E.G. McWilliam, . 2020. Organ-specific isoform selection of fatty acid-binding proteins in tissue-resident lymphocytes. Sci. Immunol. 5:eaay9283. 10.1126/sciimmunol.aay9283 32245887

[bib12] Gasteiger, G., X. Fan, S. Dikiy, S.Y. Lee, and A.Y. Rudensky. 2015. Tissue residency of innate lymphoid cells in lymphoid and nonlymphoid organs. Science. 350:981–985. 10.1126/science.aac9593 26472762 PMC4720139

[bib13] Gerlach, C., E.A. Moseman, S.M. Loughhead, D. Alvarez, A.J. Zwijnenburg, L. Waanders, R. Garg, J.C. de la Torre, and U.H. von Andrian. 2016. The chemokine receptor CX3CR1 defines three antigen-experienced CD8 T cell subsets with distinct roles in immune surveillance and homeostasis. Immunity. 45:1270–1284. 10.1016/j.immuni.2016.10.018 27939671 PMC5177508

[bib14] Guan, T., C.X. Dominguez, R.A. Amezquita, B.J. Laidlaw, J. Cheng, J. Henao-Mejia, A. Williams, R.A. Flavell, J. Lu, and S.M. Kaech. 2018. ZEB1, ZEB2, and the miR-200 family form a counterregulatory network to regulate CD8^+^ T cell fates. J. Exp. Med. 215:1153–1168. 10.1084/jem.20171352 29449309 PMC5881466

[bib15] Huang, Y., K. Mao, X. Chen, M.A. Sun, T. Kawabe, W. Li, N. Usher, J. Zhu, J.F. Urban Jr., W.E. Paul, and R.N. Germain. 2018. S1P-dependent interorgan trafficking of group 2 innate lymphoid cells supports host defense. Science. 359:114–119. 10.1126/science.aam5809 29302015 PMC6956613

[bib16] Ishii, I., N. Fukushima, X. Ye, and J. Chun. 2004. Lysophospholipid receptors: signaling and biology. Annu. Rev. Biochem. 73:321–354. 10.1146/annurev.biochem.73.011303.073731 15189145

[bib17] Jenne, C.N., A. Enders, R. Rivera, S.R. Watson, A.J. Bankovich, J.P. Pereira, Y. Xu, C.M. Roots, J.N. Beilke, A. Banerjee, . 2009. T-bet-dependent S1P5 expression in NK cells promotes egress from lymph nodes and bone marrow. J. Exp. Med. 206:2469–2481. 10.1084/jem.20090525 19808259 PMC2768857

[bib18] Joshi, N.S., W. Cui, A. Chandele, H.K. Lee, D.R. Urso, J. Hagman, L. Gapin, and S.M. Kaech. 2007. Inflammation directs memory precursor and short-lived effector CD8(+) T cell fates via the graded expression of T-bet transcription factor. Immunity. 27:281–295. 10.1016/j.immuni.2007.07.010 17723218 PMC2034442

[bib19] Kaech, S.M., and W. Cui. 2012. Transcriptional control of effector and memory CD8+ T cell differentiation. Nat. Rev. Immunol. 12:749–761. 10.1038/nri3307 23080391 PMC4137483

[bib20] Kato, Y., A. Zaid, G.M. Davey, S.N. Mueller, S.L. Nutt, D. Zotos, D.M. Tarlinton, K. Shortman, M.H. Lahoud, W.R. Heath, and I. Caminschi. 2015. Targeting antigen to Clec9A primes follicular Th cell memory responses capable of robust recall. J. Immunol. 195:1006–1014. 10.4049/jimmunol.1500767 26101322

[bib21] Kihara, Y., M. Maceyka, S. Spiegel, and J. Chun. 2014. Lysophospholipid receptor nomenclature review: IUPHAR review 8. Br. J. Pharmacol. 171:3575–3594. 10.1111/bph.12678 24602016 PMC4128058

[bib22] Kurd, N.S., Z. He, T.L. Louis, J.J. Milner, K.D. Omilusik, W. Jin, M.S. Tsai, C.E. Widjaja, J.N. Kanbar, J.G. Olvera, . 2020. Early precursors and molecular determinants of tissue-resident memory CD8^+^ T lymphocytes revealed by single-cell RNA sequencing. Sci. Immunol. 5:eaaz6894. 10.1126/sciimmunol.aaz6894 32414833 PMC7341730

[bib23] Ledgerwood, L.G., G. Lal, N. Zhang, A. Garin, S.J. Esses, F. Ginhoux, M. Merad, H. Peche, S.A. Lira, Y. Ding, . 2008. The sphingosine 1-phosphate receptor 1 causes tissue retention by inhibiting the entry of peripheral tissue T lymphocytes into afferent lymphatics. Nat. Immunol. 9:42–53. 10.1038/ni1534 18037890

[bib24] Lee, J.Y., C.N. Skon, Y.J. Lee, S. Oh, J.J. Taylor, D. Malhotra, M.K. Jenkins, M.G. Rosenfeld, K.A. Hogquist, and S.C. Jameson. 2015. The transcription factor KLF2 restrains CD4^+^ T follicular helper cell differentiation. Immunity. 42:252–264. 10.1016/j.immuni.2015.01.013 25692701 PMC4409658

[bib25] Mackay, L.K., A. Rahimpour, J.Z. Ma, N. Collins, A.T. Stock, M.L. Hafon, J. Vega-Ramos, P. Lauzurica, S.N. Mueller, T. Stefanovic, . 2013. The developmental pathway for CD103(+)CD8+ tissue-resident memory T cells of skin. Nat. Immunol. 14:1294–1301. 10.1038/ni.2744 24162776

[bib26] Mackay, L.K., A. Braun, B.L. Macleod, N. Collins, C. Tebartz, S. Bedoui, F.R. Carbone, and T. Gebhardt. 2015 a. Cutting edge: CD69 interference with sphingosine-1-phosphate receptor function regulates peripheral T cell retention. J. Immunol. 194:2059–2063. 10.4049/jimmunol.1402256 25624457

[bib27] Mackay, L.K., E. Wynne-Jones, D. Freestone, D.G. Pellicci, L.A. Mielke, D.M. Newman, A. Braun, F. Masson, A. Kallies, G.T. Belz, and F.R. Carbone. 2015 b. T-box transcription factors combine with the cytokines TGF-β and IL-15 to control tissue-resident memory T cell fate. Immunity. 43:1101–1111. 10.1016/j.immuni.2015.11.008 26682984

[bib28] Mackay, L.K., M. Minnich, N.A. Kragten, Y. Liao, B. Nota, C. Seillet, A. Zaid, K. Man, S. Preston, D. Freestone, . 2016. Hobit and Blimp1 instruct a universal transcriptional program of tissue residency in lymphocytes. Science. 352:459–463. 10.1126/science.aad2035 27102484

[bib29] Masopust, D., and A.G. Soerens. 2019. Tissue-resident T cells and other resident leukocytes. Annu. Rev. Immunol. 37:521–546. 10.1146/annurev-immunol-042617-053214 30726153 PMC7175802

[bib30] Masopust, D., D. Choo, V. Vezys, E.J. Wherry, J. Duraiswamy, R. Akondy, J. Wang, K.A. Casey, D.L. Barber, K.S. Kawamura, . 2010. Dynamic T cell migration program provides resident memory within intestinal epithelium. J. Exp. Med. 207:553–564. 10.1084/jem.20090858 20156972 PMC2839151

[bib31] Matloubian, M., C.G. Lo, G. Cinamon, M.J. Lesneski, Y. Xu, V. Brinkmann, M.L. Allende, R.L. Proia, and J.G. Cyster. 2004. Lymphocyte egress from thymus and peripheral lymphoid organs is dependent on S1P receptor 1. Nature. 427:355–360. 10.1038/nature02284 14737169

[bib32] Mayol, K., V. Biajoux, J. Marvel, K. Balabanian, and T. Walzer. 2011. Sequential desensitization of CXCR4 and S1P5 controls natural killer cell trafficking. Blood. 118:4863–4871. 10.1182/blood-2011-06-362574 21911833

[bib33] McNamara, H.A., Y. Cai, M.V. Wagle, Y. Sontani, C.M. Roots, L.A. Miosge, J.H. O’Connor, H.J. Sutton, V.V. Ganusov, W.R. Heath, . 2017. Up-regulation of LFA-1 allows liver-resident memory T cells to patrol and remain in the hepatic sinusoids. Sci. Immunol. 2:eaaj1996. 10.1126/sciimmunol.aaj1996 28707003 PMC5505664

[bib34] Milner, J.J., C. Toma, Z. He, N.S. Kurd, Q.P. Nguyen, B. McDonald, L. Quezada, C.E. Widjaja, D.A. Witherden, J.T. Crowl, . 2020. Heterogenous populations of tissue-resident CD8^+^ T cells are generated in response to infection and malignancy. Immunity. 52:808–824.e7. 10.1016/j.immuni.2020.04.007 32433949 PMC7784612

[bib35] Nüssing, S., I.G. House, C.J. Kearney, A.X.Y. Chen, S.J. Vervoort, P.A. Beavis, J. Oliaro, R.W. Johnstone, J.A. Trapani, and I.A. Parish. 2020. Efficient CRISPR/Cas9 gene editing in uncultured naive mouse t cells for in vivo studies. J. Immunol. 204:2308–2315. 10.4049/jimmunol.1901396 32152070

[bib36] Omilusik, K.D., and A.W. Goldrath. 2019. Remembering to remember: T cell memory maintenance and plasticity. Curr. Opin. Immunol. 58:89–97. 10.1016/j.coi.2019.04.009 31170601 PMC6612439

[bib37] Omilusik, K.D., J.A. Best, B. Yu, S. Goossens, A. Weidemann, J.V. Nguyen, E. Seuntjens, A. Stryjewska, C. Zweier, R. Roychoudhuri, . 2015. Transcriptional repressor ZEB2 promotes terminal differentiation of CD8+ effector and memory T cell populations during infection. J. Exp. Med. 212:2027–2039. 10.1084/jem.20150194 26503445 PMC4647262

[bib38] Park, C.O., and T.S. Kupper. 2015. The emerging role of resident memory T cells in protective immunity and inflammatory disease. Nat. Med. 21:688–697. 10.1038/nm.3883 26121195 PMC4640452

[bib39] Park, S.L., A. Zaid, J.L. Hor, S.N. Christo, J.E. Prier, B. Davies, Y.O. Alexandre, J.L. Gregory, T.A. Russell, T. Gebhardt, . 2018. Local proliferation maintains a stable pool of tissue-resident memory T cells after antiviral recall responses. Nat. Immunol. 19:183–191. 10.1038/s41590-017-0027-5 29311695

[bib40] Park, S.L., T. Gebhardt, and L.K. Mackay. 2019. Tissue-resident memory T cells in cancer immunosurveillance. Trends Immunol. 40:735–747. 10.1016/j.it.2019.06.002 31255505

[bib41] Robinette, M.L., A. Fuchs, V.S. Cortez, J.S. Lee, Y. Wang, S.K. Durum, S. Gilfillan, and M. Colonna; Immunological Genome Consortium. 2015. Transcriptional programs define molecular characteristics of innate lymphoid cell classes and subsets. Nat. Immunol. 16:306–317. 10.1038/ni.3094 25621825 PMC4372143

[bib42] Salou, M., F. Legoux, J. Gilet, A. Darbois, A. du Halgouet, R. Alonso, W. Richer, A.G. Goubet, C. Daviaud, L. Menger, . 2019. A common transcriptomic program acquired in the thymus defines tissue residency of MAIT and NKT subsets. J. Exp. Med. 216:133–151. 10.1084/jem.20181483 30518599 PMC6314520

[bib43] Sasson, S.C., C.L. Gordon, S.N. Christo, P. Klenerman, and L.K. Mackay. 2020. Local heroes or villains: tissue-resident memory T cells in human health and disease. Cell. Mol. Immunol. 17:113–122. 10.1038/s41423-019-0359-1 31969685 PMC7000672

[bib44] Schenkel, J.M., K.A. Fraser, K.A. Casey, L.K. Beura, K.E. Pauken, V. Vezys, and D. Masopust. 2016. IL-15-Independent Maintenance of tissue-resident and boosted effector memory CD8 T cells. J. Immunol. 196:3920–3926. 10.4049/jimmunol.1502337 27001957 PMC5145194

[bib45] Seki, A., and S. Rutz. 2018. Optimized RNP transfection for highly efficient CRISPR/Cas9-mediated gene knockout in primary T cells. J. Exp. Med. 215:985–997. 10.1084/jem.20171626 29436394 PMC5839763

[bib46] Sheridan, B.S., Q.M. Pham, Y.T. Lee, L.S. Cauley, L. Puddington, and L. Lefrançois. 2014. Oral infection drives a distinct population of intestinal resident memory CD8(+) T cells with enhanced protective function. Immunity. 40:747–757. 10.1016/j.immuni.2014.03.007 24792910 PMC4045016

[bib47] Shiow, L.R., D.B. Rosen, N. Brdicková, Y. Xu, J. An, L.L. Lanier, J.G. Cyster, and M. Matloubian. 2006. CD69 acts downstream of interferon-alpha/beta to inhibit S1P1 and lymphocyte egress from lymphoid organs. Nature. 440:540–544. 10.1038/nature04606 16525420

[bib48] Skon, C.N., J.Y. Lee, K.G. Anderson, D. Masopust, K.A. Hogquist, and S.C. Jameson. 2013. Transcriptional downregulation of S1pr1 is required for the establishment of resident memory CD8+ T cells. Nat. Immunol. 14:1285–1293. 10.1038/ni.2745 24162775 PMC3844557

[bib49] Slütter, B., N. Van Braeckel-Budimir, G. Abboud, S.M. Varga, S. Salek-Ardakani, and J.T. Harty. 2017. Dynamics of influenza-induced lung-resident memory T cells underlie waning heterosubtypic immunity. Sci. Immunol. 2:eaag2031. 10.1126/sciimmunol.aag2031 28783666 PMC5590757

[bib50] Stolley, J.M., T.S. Johnston, A.G. Soerens, L.K. Beura, P.C. Rosato, V. Joag, S.P. Wijeyesinghe, R.A. Langlois, K.C. Osum, J.S. Mitchell, and D. Masopust. 2020. Retrograde migration supplies resident memory T cells to lung-draining LN after influenza infection. J. Exp. Med. 217:e2092197. 10.1084/jem.20192197 PMC7398169 32568362

[bib51] Szabo, P.A., M. Miron, and D.L. Farber. 2019. Location, location, location: tissue resident memory T cells in mice and humans. Sci. Immunol. 4:eaas9673. 10.1126/sciimmunol.aas9673 30952804 PMC6778482

[bib52] Takada, K., X. Wang, G.T. Hart, O.A. Odumade, M.A. Weinreich, K.A. Hogquist, and S.C. Jameson. 2011. Kruppel-like factor 2 is required for trafficking but not quiescence in postactivated T cells. J. Immunol. 186:775–783. 10.4049/jimmunol.1000094 21160050 PMC3017213

[bib53] Takamura, S., H. Yagi, Y. Hakata, C. Motozono, S.R. McMaster, T. Masumoto, M. Fujisawa, T. Chikaishi, J. Komeda, J. Itoh, . 2016. Specific niches for lung-resident memory CD8+ T cells at the site of tissue regeneration enable CD69-independent maintenance. J. Exp. Med. 213:3057–3073. 10.1084/jem.20160938 27815325 PMC5154946

[bib54] Takamura, S., S. Kato, C. Motozono, T. Shimaoka, S. Ueha, K. Matsuo, K. Miyauchi, T. Masumoto, A. Katsushima, T. Nakayama, . 2019. Interstitial-resident memory CD8^+^ T cells sustain frontline epithelial memory in the lung. J. Exp. Med. 216:2736–2747. 10.1084/jem.20190557 31558614 PMC6888985

[bib55] Trubiano, J.A., C.L. Gordon, C. Castellucci, S.N. Christo, S.L. Park, E. Mouhtouris, K. Konvinse, M. Rose, M. Goh, A.S. Boyd, . 2020. Analysis of skin-resident memory t cells following drug hypersensitivity reactions. J. Invest. Dermatol. 140:1442–1445.e4. 10.1016/j.jid.2019.11.020 31883960 PMC7369252

[bib56] van Helden, M.J., S. Goossens, C. Daussy, A.L. Mathieu, F. Faure, A. Marçais, N. Vandamme, N. Farla, K. Mayol, S. Viel, . 2015. Terminal NK cell maturation is controlled by concerted actions of T-bet and Zeb2 and is essential for melanoma rejection. J. Exp. Med. 212:2015–2025. 10.1084/jem.20150809 26503444 PMC4647267

[bib57] Walsh, D.A., H. Borges da Silva, L.K. Beura, C. Peng, S.E. Hamilton, D. Masopust, and S.C. Jameson. 2019. The functional requirement for CD69 in establishment of resident memory CD8^+^ T cells varies with tissue location. J. Immunol. 203:946–955. 10.4049/jimmunol.1900052 31243092 PMC6684481

[bib58] Walzer, T., L. Chiossone, J. Chaix, A. Calver, C. Carozzo, L. Garrigue-Antar, Y. Jacques, M. Baratin, E. Tomasello, and E. Vivier. 2007. Natural killer cell trafficking in vivo requires a dedicated sphingosine 1-phosphate receptor. Nat. Immunol. 8:1337–1344. 10.1038/ni1523 17965716

[bib59] Watanabe, R., A. Gehad, C. Yang, L.L. Scott, J.E. Teague, C. Schlapbach, C.P. Elco, V. Huang, T.R. Matos, T.S. Kupper, and R.A. Clark. 2015. Human skin is protected by four functionally and phenotypically discrete populations of resident and recirculating memory T cells. Sci. Transl. Med. 7:279ra39. 10.1126/scitranslmed.3010302 PMC4425193 25787765

[bib60] Zhang, N., and M.J. Bevan. 2013. Transforming growth factor-β signaling controls the formation and maintenance of gut-resident memory T cells by regulating migration and retention. Immunity. 39:687–696. 10.1016/j.immuni.2013.08.019 24076049 PMC3805703

